# Supplementary material for: CRL4DCAF12 regulation of MCMBP ensures optimal licensing of DNA replication
Source: Nat Commun. 2025 Oct 27;16:9391. doi: 10.1038/s41467-025-64258-5 (PMC12559739; doi:10.1038/s41467-025-64258-5)
Supplement: Supplementary file 1 — Supplementary Information [file 41467_2025_64258_MOESM1_ESM.pdf]

SUPPLEMENTARY INFORMATION

CRL4^{DCAF12} regulation of MCMBP ensures optimal licensing of DNA replication

Anoop Kumar Yadav[1,2+], Alikhan Abdirov[3+], Katarina Ondruskova[3], Simran Negi[1,2], Kristina Jamrichova[3], Karolina Kolarova[3,4], Nikol Dibus[3], Jana Krejci[1], Hana Polasek-Sedlackova[1*] and Lukas Cermak[3*]

[1]*Institute of Biophysics, Czech Academy of Sciences, Brno, Czech Republic*

[2]*Department of Experimental Biology, Faculty of Science, Masaryk University, Brno, Czech Republic*

[3]*Laboratory of Cancer Biology, Institute of Molecular Genetics of the Czech Academy of Sciences, Prague, Czech Republic*

[4]*Faculty of Science, Charles University, Prague, Czech Republic*

[+]These authors contributed equally.

[*]Corresponding authors: H.P.-S. (polasek-sedlackova@ibp.cz), L.C. (lukas.cermak@img.cas.cz)

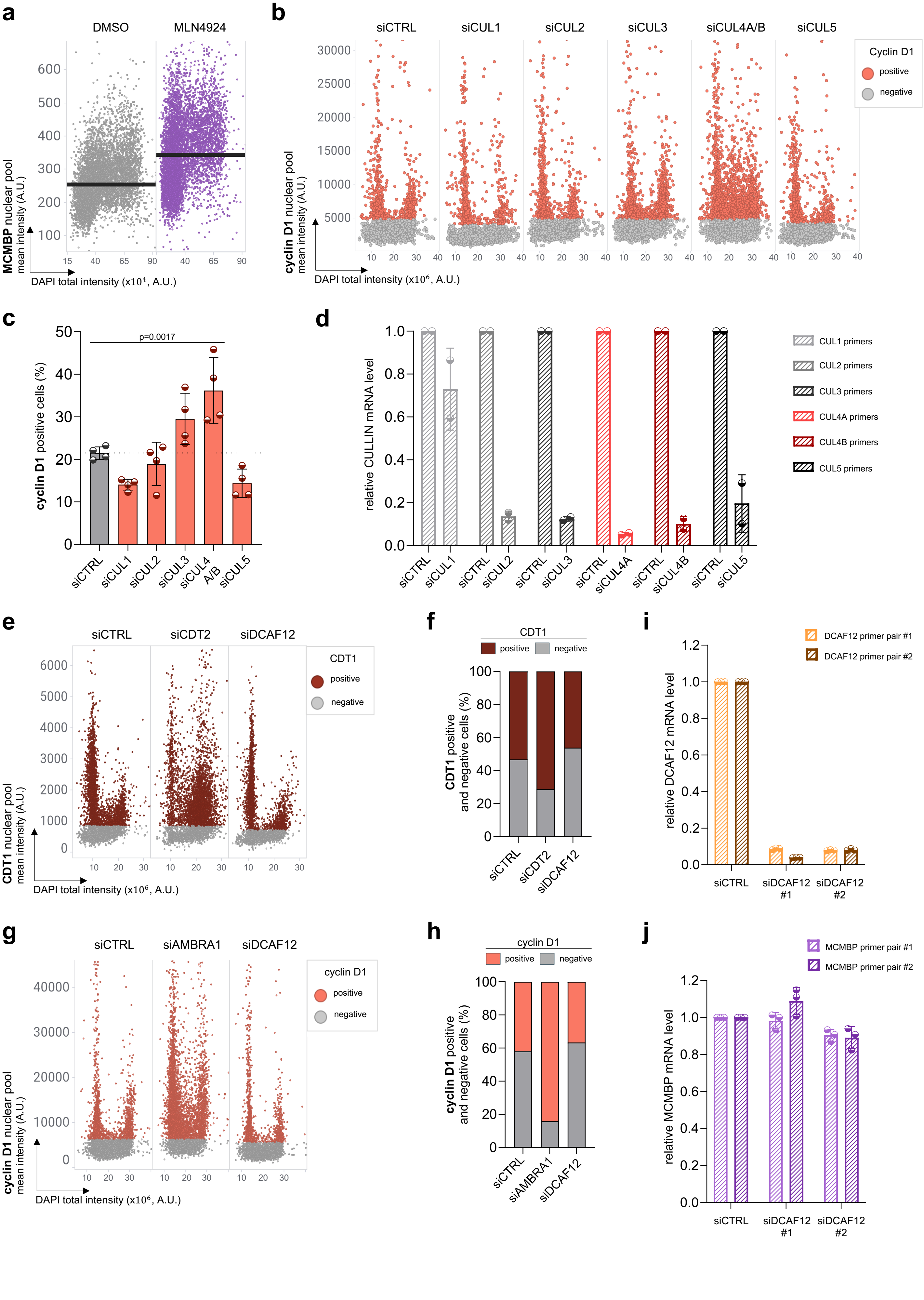

Supplementary Fig. 1

Supplementary Fig. 1: MLN4924, a protein neddylation inhibitor, increases cellular levels of MCMBP. a, QIBC plots of total nuclear MCMBP in U2OS cells after treatment with DMSO or MLN4924 (5 µM, 6 h). Lines denote medians; n = 6,844 (minimum cells per condition). **b,** QIBC plots of MCM4-Halo cells immunostained for cyclin D1 after indicated siRNA treatments; n = 4,929 (minimum cells per condition). **c,** Quantification of cyclin D1 positive cells (based on sub-stratification in **b**). Each bar indicates the percentage of cyclin D1-positive cells (data are mean ± s.d.; n = 4 technical replicates). **d**, RT-PCR-based analysis of relative mRNA levels of cullins in cells treated with control siRNA (siCTRL) or siRNA against respective cullins (as indicated); normalized with respect to siCTRL cells. n = 2 technical replicates. **e,** QIBC plots of MCM4-Halo cells immunostained for CDT1 after indicated siRNA treatments; n = 4,915 (minimum cells per condition). **f,** Quantification of QIBC plots in **e**; each bar indicates the percentage distribution of CDT1 positive and negative cells (based on sub-stratification in **e**). **g,** QIBC of MCM4-Halo cells immunostained for cyclin D1 after indicated siRNA treatments; n = 5,013 (minimum cells per condition). **h,** Quantification of QIBC plots in **g**; each bar indicates the percentage distribution of cyclin D1 positive and negative cells (based on sub-stratification in **g**). **i,** qPCR-based analysis of DCAF12 mRNA levels after indicated siRNA treatments; normalized with respect to siCTRL (data are mean ± s.d.; n = 3 technical replicates). **j,** qPCR-based analysis of MCMBP mRNA levels upon indicated siRNA treatments; normalized with respect to siCTRL treatment (data are mean ± s.d.; n = 3 technical replicates). P values were calculated by ordinary one-way ANOVA with Dunnett's test (**c**). A.U. Arbitrary Units.

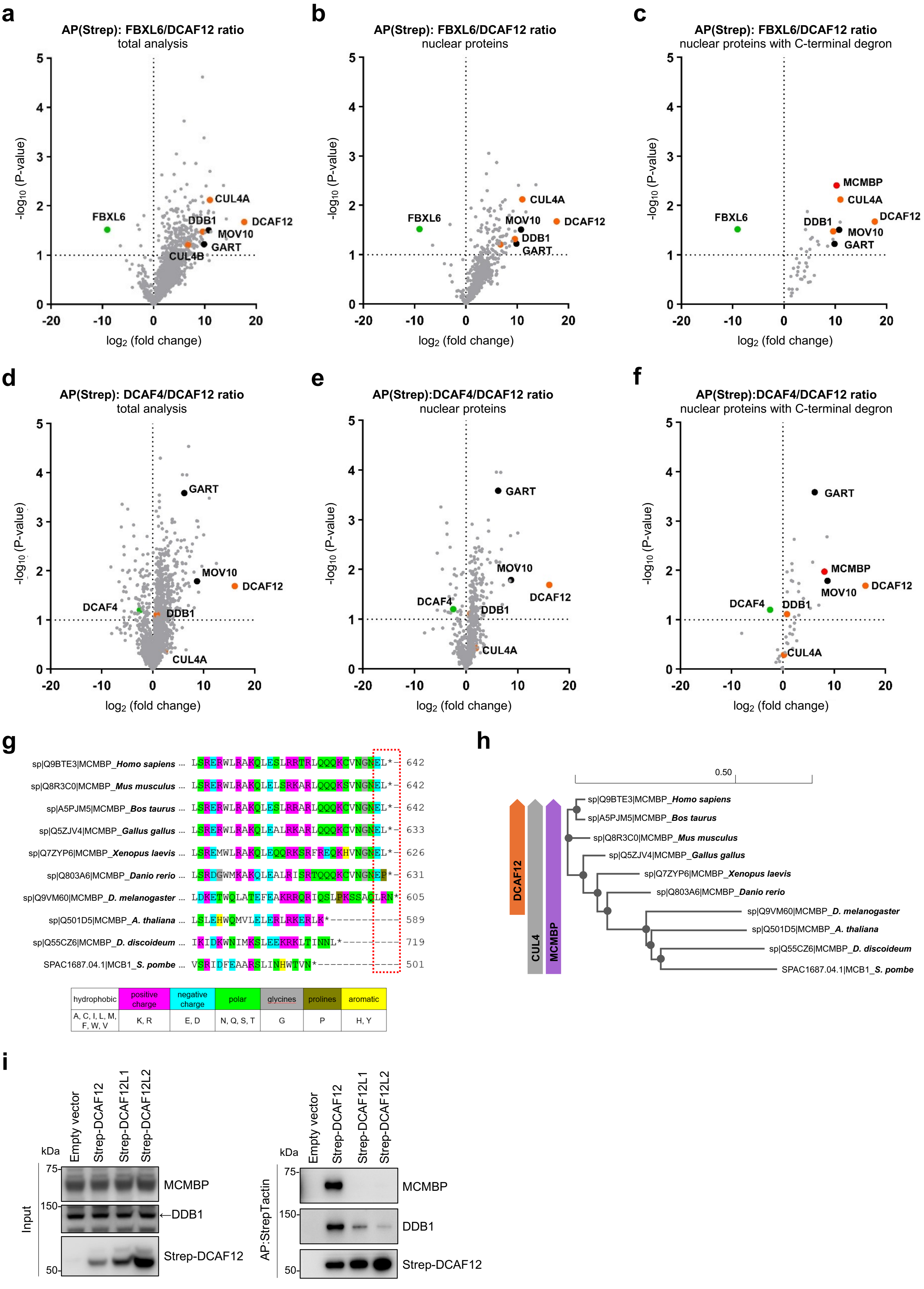

Supplementary Fig. 2

Supplementary Fig. 2: MCMBP specifically interacts with DCAF12 but not with its mammalian paralogs. **a,** $CRL4^{DCAF12}$-associated proteins identified by liquid chromatography-tandem mass spectrometry (LC-MS/MS). The composition of purified complexes from both steps was analyzed by LC-MS/MS. Non-related ubiquitin ligase SCF^{FBXL6} was used as a control. Proteins that were significantly enriched with $CRL4^{DCAF12}$ compared to SCF^{FBXL6} are in the upper right quadrant. **b,** Same as in a, but only nuclear proteins are shown. **c,** Same as in a, but only nuclear proteins with C-terminal acidic degron are shown. **d,** $CRL4^{DCAF12}$-associated proteins identified by liquid chromatography-tandem mass spectrometry (LC-MS/MS). The composition of purified complexes from both steps was analyzed by LC-MS/MS. Non-related ubiquitin ligase $CRL4^{DCAF4}$ was used as a control. Proteins that were significantly enriched with $CRL4^{DCAF12}$ compared to $CRL4^{DCAF4}$ are in the upper right quadrant. **e,** Same as in a, but only nuclear proteins are shown **f,** Same as in a, but only nuclear proteins with C-terminal acidic degron are shown. **g,** Multiple sequence alignment of the C-terminal sequence of MCMBP protein from indicated species. Sequences were aligned using EMBL-EBI Clustal Omega[43]. **h,** Phylogram of species used in multiple sequence alignment in **g** with indicated emergence of *DCAF12*, *CUL4*, and *MCMBP* genes. **i,** Affinity purification of N-terminal STREP-FLAG-tagged DCAF12 and its paralogs. HEK293T cells were co-transfected with StrepII-FLAG-tagged DCAF12, DCAF12L1 and DCAF12L2. MLN4924 was added for the final 6 hours before harvest. Following lysis, whole-cell lysates (WCL) were subjected to affinity purification (AP) with Strep-TactinXT resin and immunoblotted as indicated. The left panel shows 1% input samples, the right panel shows Strep-Tactin AP. n = 1 biological replicate.

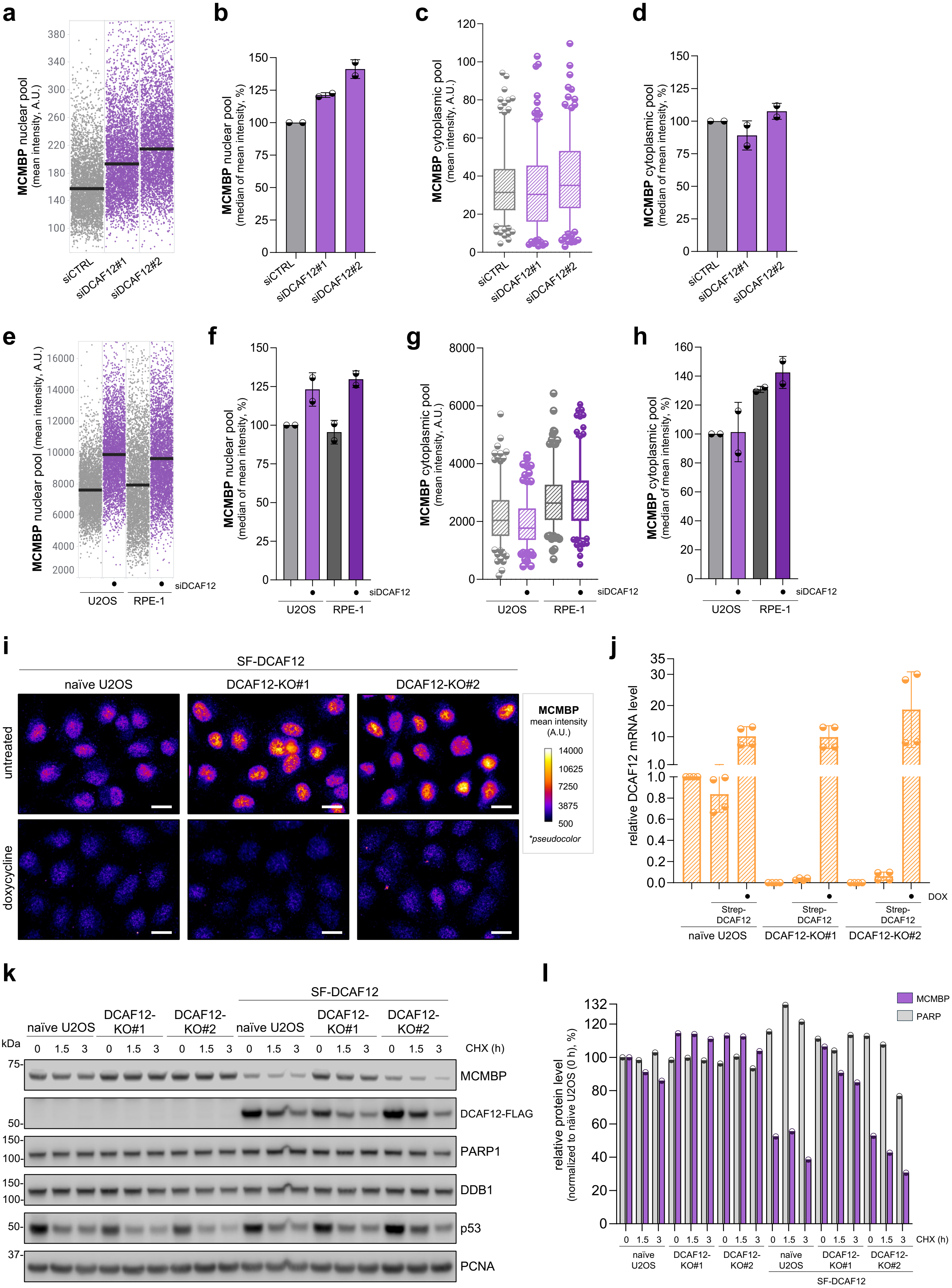

Supplementary Fig. 3

Supplementary Fig. 3: Depletion of DCAF12 leads to stabilization of MCMBP protein levels in the nucleus. a, QIBC plots of MCM4-Halo cells immunostained for MCMBP after treatment with control siRNA or siRNAs targeting different regions of the *DCAF12* gene (siDCAF12#1 or siDCAF12#2), as indicated. Lines denote medians; n = 4,334 (minimum cells per condition). **b,** Quantification of QIBC plots in **a**, each bar indicates the median of mean intensity normalized with respect to siCTRL as 100 percent (data are mean ± s.d.; n = 2 biological replicates). **c,** MFI of cytoplasmic MCMBP after indicated siRNA treatments. **d,** Quantification of MFI of cytoplasmic MCMBP in **c**; each bar indicates the median of mean intensity normalized with respect to siCTRL as 100 percent (data are mean ± s.d.; n = 2 biological replicates). **e,** QIBC of U2OS and RPE-1 cells immunostained for MCMBP upon siRNA treatment against DCAF12, as indicated. Lines denote medians; n = 4,029 (minimum cells per condition). **f,** Quantification of QIBC in **e**, each bar indicates the median of mean intensity normalized with respect to untreated U2OS cells as 100 percent (data are mean ± s.d.; n = 2 technical replicates). **g,** MFI of cytoplasmic MCMBP under similar conditions as in **e**. **h,** Quantification of the MFI of cytoplasmic MCMBP in **g**; each bar indicates the median of mean intensity normalized with respect to untreated U2OS cells as 100 percent (data are mean ± s.d.; n = 2 technical replicates). **i,** Representative images of naïve U2OS cells and DCAF12 knock-out U2OS cells (DCAF12-KO#1, DCAF12-KO#2) expressing doxycycline (DOX, 48 hours) inducible Strep-DCAF12 (where indicated) immunostained for MCMBP (without pre-extraction); the pseudo-color gradient indicates the MFI. Scale bar, 20 μm. **j**, RT-PCR-based analysis of DCAF12 mRNA levels in naïve U2OS cells and DCAF12 knock-out U2OS cells (DCAF12-KO#1, DCAF12-KO#2) expressing DOX inducible Strep-DCAF12 (where indicated); normalized with respect to naïve U2OS cells. n = 4 (2 biological replicates, each with 2 technical replicates). **k**, StrepII-FLAG-tagged DCAF12 (SF-DCAF12) was inducible expressed in DCAF12 WT and KO U2OS cells using the Sleeping Beauty Transposon System. Where indicated, the expression of DCAF12 was induced with doxycycline for 24 h. Cells were treated with cycloheximide (CHX) for the indicated time. Soluble protein extracts were immunoblotted as indicated**. l,** Quantification of MCMBP staining in **k,** each bar indicates relative protein levels normalized with respect to naïve U2OS (0h) as 100 percent. In box plots (**c, g**), central lines denote medians, the boxes indicate the 25th and 75th centiles, the whiskers indicate 5 and 95 percentile values; n = 200 cells per condition. Data are absolute values from n = 1 biological replicate.

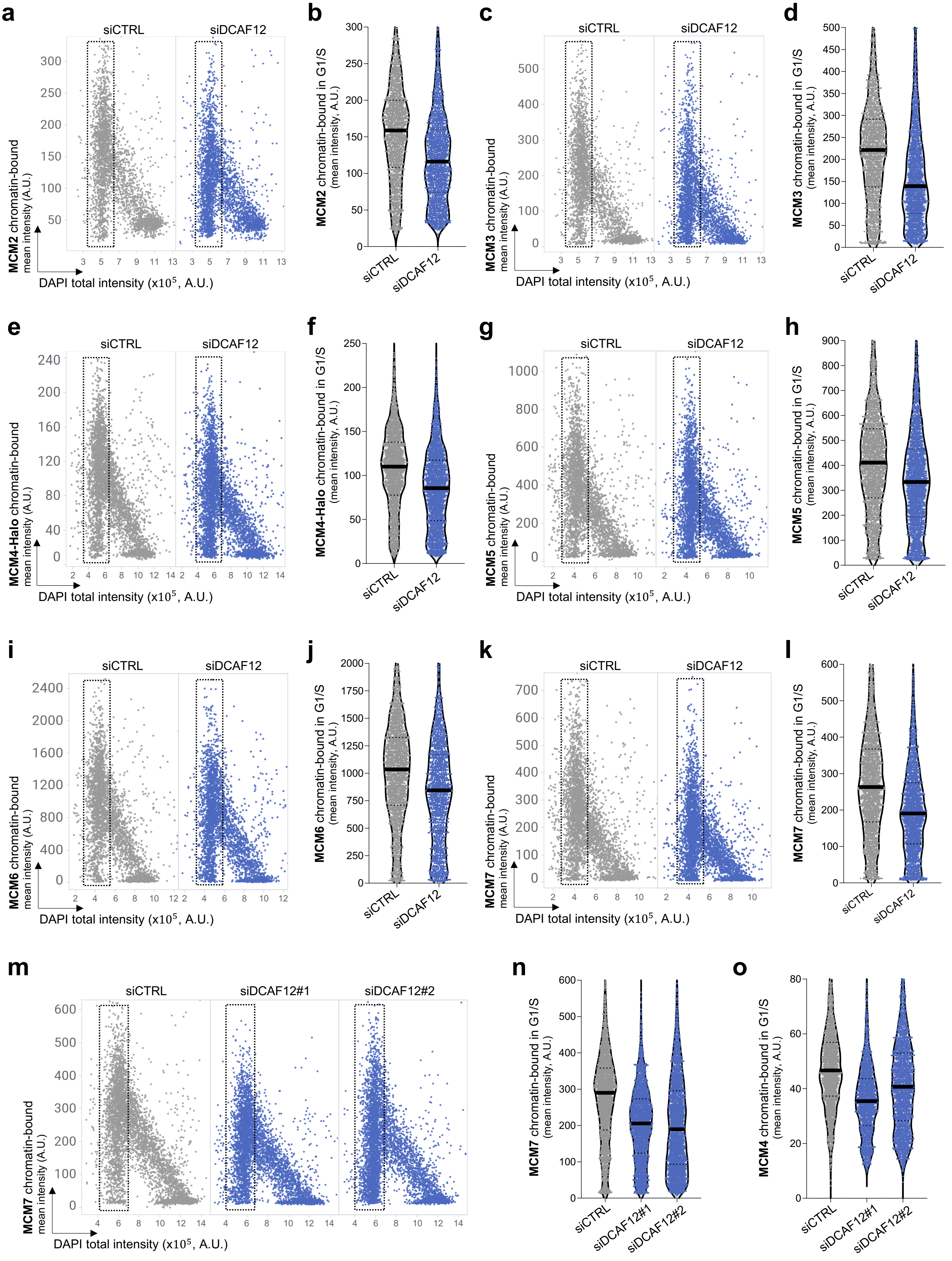

Supplementary Fig. 4

Supplementary Fig. 4: Downregulation of DCAF12 results in reduced chromatin binding of all MCM subunits. a, QIBC plots of pre-extracted U2OS cells stained for MCM2 after treatment with control siRNA or siRNA against DCAF12. DAPI counterstains nuclear DNA. n = 3,473 (minimum cells per condition). Dashed boxes mark the G1/S phase stage. **b,** MFI of MCM2 in G1/S cells based QIBC in **a**. Lines denote medians; n = 1,694 (minimum cells per condition). **c,** QIBC plots of pre-extracted U2OS cells stained for MCM3 after treatment as indicated. n = 3,473 (minimum cells per condition). **d,** MFI of MCM3 in G1/S cells based QIBC in **c**. Lines denote medians; n = 1,694 (minimum cells per condition). **e,** QIBC plots of pre-extracted U2OS cells stained for MCM4 after treatment as indicated. n = 4,121 (minimum cells per condition). **f,** MFI of MCM4 in G1/S cells based QIBC in **e**. Lines denote medians; n = 2,103 (minimum cells per condition). **g,** QIBC plots of pre-extracted U2OS cells stained for MCM5 after treatment as indicated. n = 4,062 (minimum cells per condition). **h,** MFI of MCM5 in G1/S cells based QIBC in **g**. Lines denote medians; n = 2,103 (minimum cells per condition). **i,** QIBC plots of pre-extracted U2OS cells stained for MCM6 after treatment as indicated. n = 3,525 (minimum cells per condition). **j,** MFI of MCM6 in G1/S cells based QIBC in **i**. Lines denote medians; n = 1,802 (minimum cells per condition). **k,** QIBC plots of pre-extracted U2OS cells stained for MCM7 after treatment as indicated. n = 4,062 (minimum cells per condition). **l,** MFI of MCM7 in G1/S cells based QIBC in **k**. Lines denote medians; n = 2,103 (minimum cells per condition). **m,** QIBC plots of pre-extracted U2OS cells stained for MCM7 after treatment as indicated. n = 5,015 (minimum cells per condition). **n,** MFI of MCM7 in G1/S cells based QIBC in **m**. Lines denote medians; n = 2,352 (minimum cells per condition). **o,** MFI of MCM4 in G1/S cells after treatment as indicated. Lines denote medians; n = 1,786 (minimum cells per condition). A.U. Arbitrary Units.

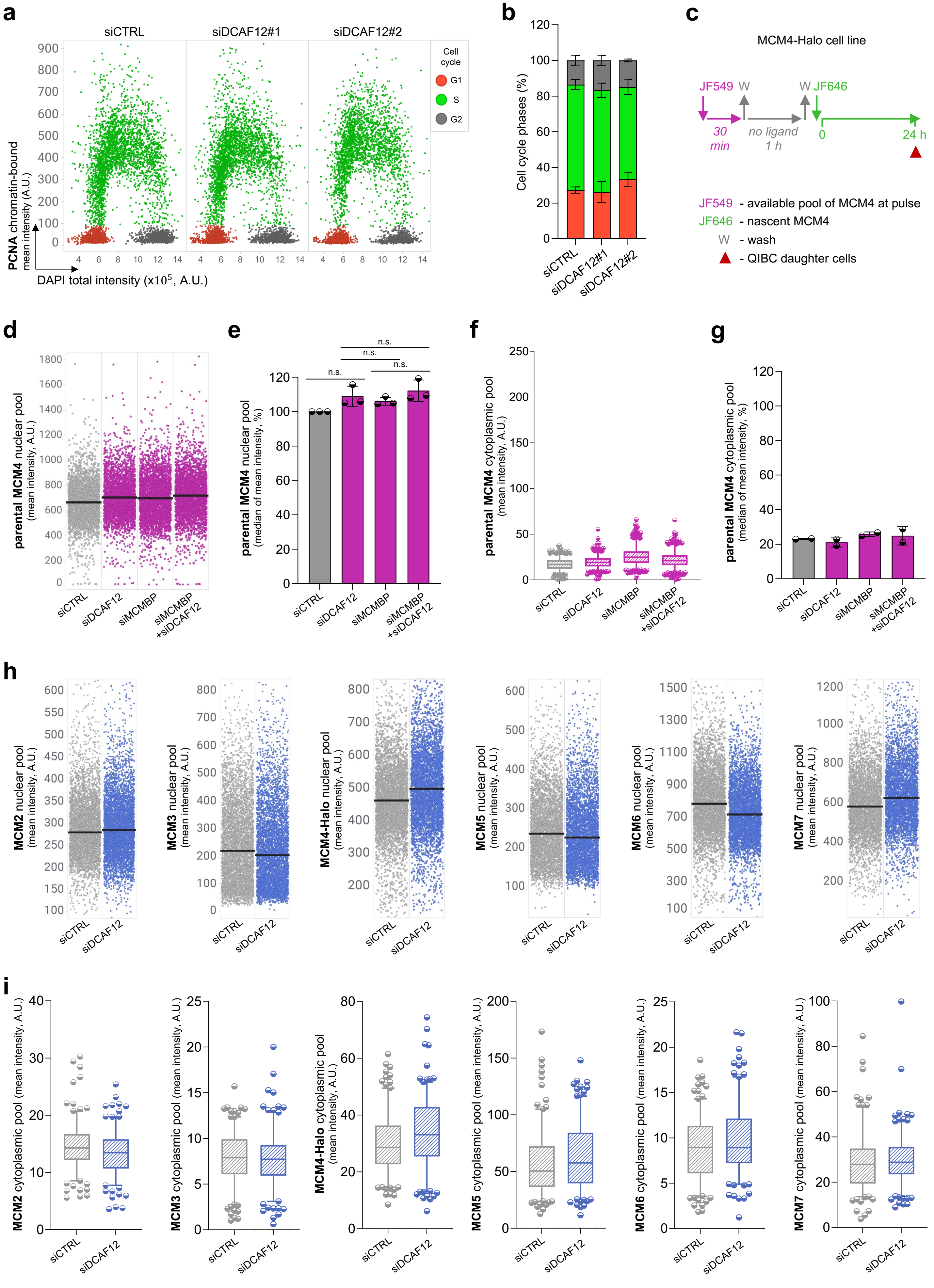

Supplementary Fig. 5

Supplementary Fig. 5: Nuclear levels or localization of MCM subunits are not affected upon DCAF12 depletion. a, QIBC plots of pre-extracted U2OS cells stained for PCNA after treatment with control siRNA or two different siRNAs against DCAF12, as indicated. DAPI counterstains nuclear DNA; n = 5,015 (minimum cells per condition). **b,** Quantification of individual cell cycle phases based on QIBC in **a**; n = 2 biological replicates. **c,** Dual HaloTag labeling protocol in U2OS cells expressing endogenously tagged MCM4-Halo. **d,** QIBC plots of MCM4-Halo U2OS cells stained for parental MCM4 after indicated siRNA treatments. Lines denote medians; n = 3,351 (minimum cells per condition). **e,** Quantification of QIBC plots in **d**; each bar indicates the median of mean intensity (data are mean ± s.d.; n = 3 biological replicates). **f,** MFI of cytoplasmic parental MCM4 after indicated siRNA treatments. **g,** Quantification of MFI of cytoplasmic parental MCM4; each bar indicates the median of mean intensity (data are mean ± s.d.; n = 3 biological replicates). **h,** QIBC plots of U2OS cells stained for MCM2, MCM3, MCM4-Halo, MCM5, MCM6 and MCM7, respectively; after indicated siRNA treatments. Lines denote medians; n = 5,124 (minimum cells per condition, MCM2, MCM3); n = 4,534 (minimum cells per condition, MCM4-Halo, MCM6), n = 4,545 (minimum cells per condition, MCM5, MCM7). **i,** MFI of cytoplasmic MCM2, MCM3, MCM4, MCM5, MCM6 and MCM7, respectively; after indicated siRNA treatments. In box plots (**i**), central lines denote medians, the boxes indicate the 25th and 75th centiles, the whiskers indicate 5 and 95 percentile values; n = 200 cells per condition. P values were calculated by ordinary one-way ANOVA with Šidák's test (**e**); n.s. indicates p>0.1. A.U. Arbitrary Units.

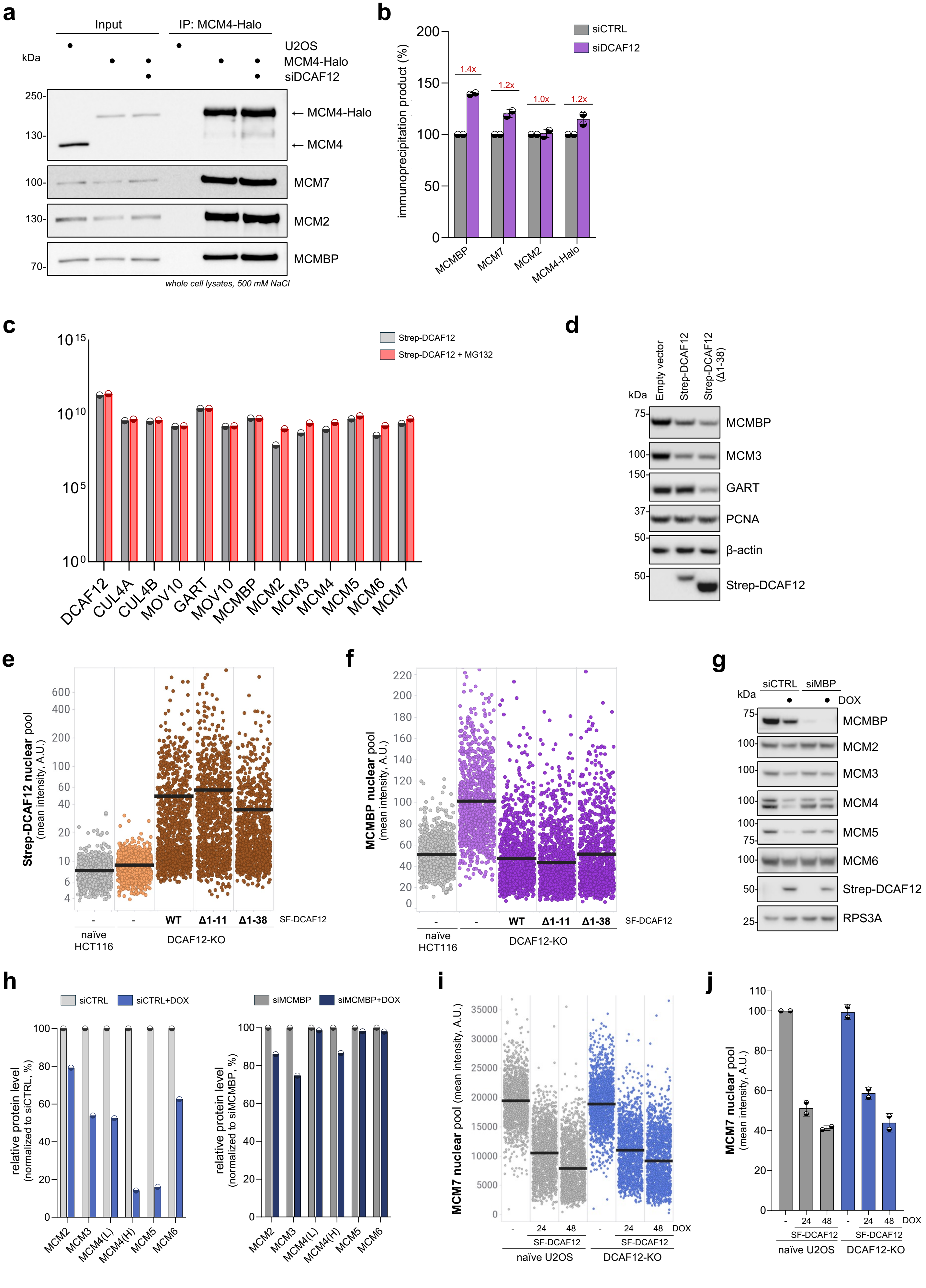

Supplementary Fig. 6

Supplementary Fig. 6: Precise cellular levels of DCAF12 are critical to maintain MCMBP levels and optimal MCM equilibrium. a, Halo-immunoprecipitation (Halo-IP) of whole cell lysates followed by western blotting, cells were treated with siRNA against DCAF12 (siDCAF12) as indicated. This IP was done in high salt condition (500 mM NaCl). **b,** Quantification of Halo-IP products based on western blots in **a**; IP products upon control siRNA treatment have been considered as 100% (data are mean ± s.d.; n = 2 biological replicates). **c**, Bar graph, derived from mass spectrometry analysis of HEK293T cells co-transfected with StrepII-FLAG-tagged DCAF12 in Fig. 5j, shows the intensities of indicated proteins interacting with DCAF12 in untreated and MG132-treated samples. Cells were treated with either DMSO or 10 μM MG132 for 6 hours prior to protein purification. **d,** StrepII-FLAG-tagged DCAF12 (SF-DCAF12) or its mutant lacking NLS was inducibly expressed in HCT116 cells using the Sleeping Beauty Transposon System. Where indicated, the expression of constructs was induced with doxycycline for 24 h. Whole-cell lysates were immunoblotted as indicated. n = 1 biological replicate. **e, f,** QIBC plots of HCT116 cells nuclei stained for StrepII-tagged DCAF12 (**e**) or MCMBP (**f**) protein after indicated DCAF12 constructs were inducibly expressed. Lines denote medians; n = 1,051 in (**e**) and 1,070 in (**f**) minimum cells per condition. **g**, StrepII-FLAG-tagged DCAF12 was inducibly expressed in U2OS cells using the Sleeping Beauty Transposon System. Where indicated, the expression of DCAF12 was induced with doxycycline for 24 h. Cells were transfected with control or MCMBP (MBP) targeting siRNA. Whole-cell lysates were immunoblotted as indicated. **h,** Densitometry of selected protein staining in **g; i,** QIBC plots of naïve or DCAF12 knock-out (DCAF12-KO) U2OS cells transfected with doxycycline (DOX) inducible SF-DCAF12 and immunostained for total nuclear MCM7 after 0, 24 and 48 hours of DOX treatment. Lines denote medians; n = 1,503 (minimum cells per condition). Data are absolute values from n = 1 biological replicate. **j,** Quantification of QIBC plots in **i**; each bar indicates the median of mean intensity (data are mean ± s.d.; n = 2 technical replicates). A.U. Arbitrary Units.

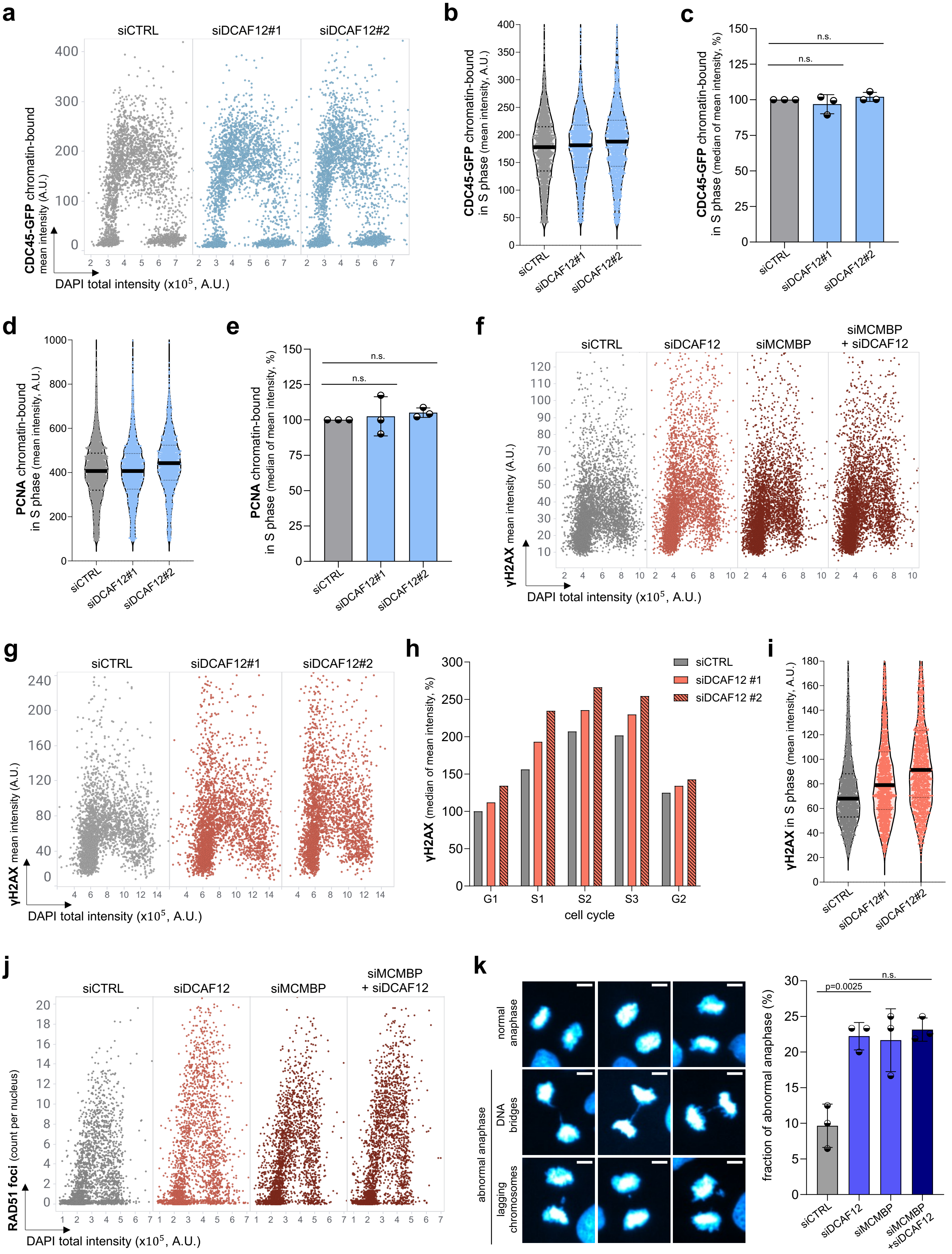

Supplementary Fig. 7

Supplementary Fig. 7: CRL4^{DCAF12}-dependent regulation of MCMBP preserves genome stability. a, QIBC plots of chromatin-bound CDC45-GFP in CDC45-GFP cells treated with control siRNA or siRNAs targeting different regions of the *DCAF12* gene (siDCAF12#1 or siDCAF12#2), as indicated. DAPI counterstains nuclear DNA. Lines denote medians; n = 3,531 (minimum cells per condition). **b,** MFI of chromatin-bound CDC45-GFP in S phase cells based on QIBC plots in **a.** Lines denote medians; n = 1,985 (minimum cells per condition). **c,** Quantification of the MFI of CDC45-GFP in S phase cells based on QIBC plots in **a**. Each bar indicates the median of mean intensity normalized with respect to siCTRL as 100 percent; data are mean ± s.d.; n = 3 biological replicates. **d,** MFI of chromatin-bound PCNA in S phase cells based on QIBC in Supplementary Fig. 5a. Lines denote medians; n = 2,415 (minimum cells per condition). **e,** Quantification of the MFI of PCNA in S phase cells based on QIBC in Supplementary Fig. 5a. Each bar indicates the median of mean intensity normalized with respect to siCTRL as 100 percent; data are mean ± s.d.; n = 3 biological replicates. **f,** QIBC plots of U2OS cells immunostained for γH2AX after indicated siRNA treatments. n = 4,714 (minimum cells per condition). **g,** QIBC plots of U2OS cells immunostained for γH2AX after siRNA treatment as indicated. n = 3,009 (minimum cells per condition). **h,** Quantification of the level of γH2AX at indicated stages of the cell cycle based on QIBC plots in **g**. Each bar indicates the median of mean intensity normalized with respect to G1 in siCTRL as 100 percent. **i,** MFI of γH2AX in S phase cells based on QIBC plots in **g**. Lines denote median, n = 1,526 (minimum cells per condition). **j,** QIBC plots of RAD51 foci count per nucleus after indicated siRNA treatments, n = 2,790 (minimum cells per condition). **k,** Left, representative images of anaphase anomalies such as DNA bridges and lagging chromosomes. Right, quantification of the percentage of abnormal anaphases observed after indicated siRNA treatments. Data are mean ± s.d.; n = 3 biological replicates. P values were calculated by ordinary one-way ANOVA with Šidák's test (**c, e, k**); n.s. indicates p>0.1. A.U. Arbitrary Units.
