# Supplementary material for: CRL4DCAF12 regulation of MCMBP ensures optimal licensing of DNA replication
Source: Nat Commun. 2025 Oct 27;16:9391. doi: 10.1038/s41467-025-64258-5 (PMC12559739; doi:10.1038/s41467-025-64258-5)
Supplement: Supplementary file 2 — Reporting Summary [file 41467_2025_64258_MOESM2_ESM.pdf]

nature portfolio

Corresponding author(s): Hana Polasek-Sedlackova, Lukas Cermak

Last updated by author(s): Aug 20, 2025

Reporting Summary

Nature Portfolio wishes to improve the reproducibility of the work that we publish. This form provides structure for consistency and transparency in reporting. For further information on Nature Portfolio policies, see our Editorial Policies and the Editorial Policy Checklist.

Statistics

For all statistical analyses, confirm that the following items are present in the figure legend, table legend, main text, or Methods section.

n/a	Confirmed	
☐	☒	The exact sample size (*n*) for each experimental group/condition, given as a discrete number and unit of measurement
☐	☒	A statement on whether measurements were taken from distinct samples or whether the same sample was measured repeatedly
☐	☒	The statistical test(s) used AND whether they are one- or two-sided<br>*Only common tests should be described solely by name; describe more complex techniques in the Methods section.*
☒	☐	A description of all covariates tested
☒	☐	A description of any assumptions or corrections, such as tests of normality and adjustment for multiple comparisons
☐	☒	A full description of the statistical parameters including central tendency (e.g. means) or other basic estimates (e.g. regression coefficient) AND variation (e.g. standard deviation) or associated estimates of uncertainty (e.g. confidence intervals)
☐	☒	For null hypothesis testing, the test statistic (e.g. *F*, *t*, *r*) with confidence intervals, effect sizes, degrees of freedom and *P* value noted<br>*Give P values as exact values whenever suitable.*
☒	☐	For Bayesian analysis, information on the choice of priors and Markov chain Monte Carlo settings
☒	☐	For hierarchical and complex designs, identification of the appropriate level for tests and full reporting of outcomes
☒	☐	Estimates of effect sizes (e.g. Cohen's *d*, Pearson's *r*), indicating how they were calculated

Our web collection on statistics for biologists contains articles on many of the points above.

Software and code

Policy information about availability of computer code

Data collection	ScanR acquisition software (Evident, v.3.4.1) and ScanR acquisition software (Evident, v.2.7.1) for ScanR high-content microscopy
Data analysis	ScanR analysis software (Evident, v.3.4.1, for QIBC)<br>Excel (Microsoft, v.2409 build 16.0.18025.20030, for image analysis data management)<br>Spotfire (TIBCO, v.11.8.0, for data visualization)<br>GraphPad Prism (v.10.1.2, for data visualization and statistics)<br>ImageJ (FIJI, v.1.54i, cytoplasmic level analysis)<br>MaxQuant (version 1.5.3.8, mass-spectrometry data analysis (Fig. 1h-i))<br>MaxQuant (version 2.3.0.0, mass-spectrometry data analysis (Fig. 5j))<br>Perseus (version 1.5.2.4, statistical analysis of mass-spectrometry data (Fig. 1h-i))<br>Perseus (version 1.6.15.0, statistical analysis of mass-spectrometry data (Fig. 5j))

For manuscripts utilizing custom algorithms or software that are central to the research but not yet described in published literature, software must be made available to editors and reviewers. We strongly encourage code deposition in a community repository (e.g. GitHub). See the Nature Portfolio guidelines for submitting code & software for further information.

Data

Policy information about availability of data

All manuscripts must include a data availability statement. This statement should provide the following information, where applicable:

- Accession codes, unique identifiers, or web links for publicly available datasets
- A description of any restrictions on data availability
- For clinical datasets or third party data, please ensure that the statement adheres to our policy

There are no restrictions on data availability. All the source data, including numerical and statistical source data, uncropped scans of all blots and gels, are provided with this paper. The proteomic datasets are deposited at the ProteomeXchange database with identifiers PXD055947 [https://proteomecentral.proteomexchange.org/cgi/GetDataset?ID=PXD055947] and PXD067954 [https://proteomecentral.proteomexchange.org/cgi/GetDataset?ID=PXD067954].

Research involving human participants, their data, or biological material

Policy information about studies with human participants or human data. See also policy information about sex, gender (identity/presentation), and sexual orientation and race, ethnicity and racism.

Reporting on sex and gender	Not applicable, because this study does not involve human research participants.
Reporting on race, ethnicity, or other socially relevant groupings	Not applicable, because this study does not involve human research participants.
Population characteristics	Not applicable, because this study does not involve human research participants.
Recruitment	Not applicable, because this study does not involve human research participants.
Ethics oversight	Not applicable, because this study does not involve human research participants.

Note that full information on the approval of the study protocol must also be provided in the manuscript.

Field-specific reporting

Please select the one below that is the best fit for your research. If you are not sure, read the appropriate sections before making your selection.

☒ Life sciences ☐ Behavioural & social sciences ☐ Ecological, evolutionary & environmental sciences

For a reference copy of the document with all sections, see nature.com/documents/nr-reporting-summary-flat.pdf

Life sciences study design

All studies must disclose on these points even when the disclosure is negative.

Sample size	No statistical methods were used to predetermine sample size. For all the experiments, the sample sizes were determined based on experimental experience, technical expertise and currently accepted standards in the field of high-content imaging (Toledo et al., Cell 2013; Somyajit et al, Science 2017; Sedlackova et al. Nature 2020) and DNA fiber analysis (Somyajit et al, Science 2017; Sedlackova et al. Nature 2020).
Data exclusions	All data acquired for this study were included in the analysis.
Replication	All experimental findings were reliably reproduced as indicated in the figure legends. All attempts at replication were successful.
Randomization	Samples were organized into groups based on treatments (e.g., untreated vs. treatment; control siRNA treated compared to target-specific siRNAs; experimental time-points).<br>Cell cultures within control variables or experimental groups were randomly assigned. Appropriate controls were included in all the experiments.
Blinding	For QIBC, images were acquired and analyzed in an unbiased manner using automated acquisition and analysis software, rendering blinding moot.

Reporting for specific materials, systems and methods

We require information from authors about some types of materials, experimental systems and methods used in many studies. Here, indicate whether each material, system or method listed is relevant to your study. If you are not sure if a list item applies to your research, read the appropriate section before selecting a response.

Materials & experimental systems

n/a	Involved in the study
☐	☒ Antibodies
☐	☒ Eukaryotic cell lines
☒	☐ Palaeontology and archaeology
☒	☐ Animals and other organisms
☒	☐ Clinical data
☒	☐ Dual use research of concern
☒	☐ Plants

Methods

n/a	Involved in the study
☒	☐ ChIP-seq
☒	☐ Flow cytometry
☒	☐ MRI-based neuroimaging

Antibodies

Antibodies used

Primary antibodies used for immunofluorescence (IF) were as follows:
CDT1 (rabbit, Abcam, ab202067, 1:2,000; Validation: manufacturers website); Cyclin D1 (rabbit, Proteintech, 26939-1-AP, 1:1,000; Validation: manufacturers website); GFP (rabbit, Proteintech, PABG1, 1:5,000; Validation: manufacturers website); MCMBP (rabbit, Novus Biologicals, NBP1-90746, 1:1,000; Validation: manufacturers website); MCM2 (rabbit, Proteintech, 10513-1-AP, 1:1,000; Validation: manufacturers website and this study); MCM3 (mouse, Santa Cruz, sc-390480, 1:1,000; Validation: manufacturers website and this study); MCM4 (rabbit, Proteintech, 13043-1-AP, 1:1,000; Validation: manufacturers website and this study); MCM5 (rabbit, Proteintech, 11703-1-AP, 1:1,000; Validation: manufacturers website and this study); MCM6 (mouse, Novus Biologicals, H00004175-M04, 1:1,000; Validation: manufacturers website and this study); MCM7 (mouse, Santa Cruz, sc-9966, 1:1,000; Validation: manufacturers website and this study); PCNA (human, Immuno Concepts, 2037, 1:1,000; Validation: validated for IF experiments by QIBC in previous studies e.g. Somyajit et al, Science 2017; Polasek-Sedlackova et al, Nat Commun 2022); RAD51 (rabbit, BioAcademia, 70-012, 1:1,000; Validation: manufacturers website); Strep II Tag (mouse, Novus Biologicals, NBP2-43735, 1:1,000; Validation: manufacturers website); γH2AX (Ser139) (rabbit, Abcam, ab81299, 1:1,000, Validation: manufacturers website).

Primary antibodies used for western blotting were as follows:
α-tubulin (mouse, Proteintech, 66031-1-Ig, 1:1,000; Validation: manufacturers website); β-actin (mouse, Santa Cruz, sc-69879, 1:1,000; Validation: manufacturers website), DDB1 (rabbit, Zymed, 34-2300, 1:1,000; Validation: manufacturers website), GART (mouse, Santa Cruz, sc-166379, 1:1,000; Validation: manufacturers website); γH2AX (rabbit, Proteintech, 83307-2-RR; Validation: manufacturers website); γH2AX (mouse, Milipore, 05-636, 1:1,000; Validation: manufacturers website); HA (rabbit, Cell Signaling, 3724, 1:1,000; Validation: manufacturers website); MCMBP (rabbit, Proteintech, 19573-1-AP, 1:1,000; Validation: manufacturers website); MCMBP (rabbit, Atlas Antibodies, HPA038481, 1:1,000; Validation: manufacturers website); MCM2 (rabbit, Proteintech, 10513-1-AP, 1:1,000; Validation: manufacturers website and this study); MCM2 (rabbit, ABClonal, A1056, 1:1,000; Validation: manufacturers website); MCM3 (rabbit, ABClonal, A1060, 1:1,000; Validation: manufacturers website); MCM4 (rabbit, Proteintech, 13043-1-AP, 1:1,000; Validation: manufacturers website and this study); MCM5 (rabbit, Proteintech, 11703-1-AP, 1:1,000; Validation: manufacturers website and this study); MCM6 (rabbit, Proteintech, 13347-2-AP, 1:1,000; Validation: manufacturers website and this study); MCM7 (mouse, Santa Cruz, sc-9966, 1:1,000; Validation: manufacturers website and this study); MCM7 (rabbit, Proteintech, 11225-1-AP, 1:1,000; Validation: manufacturers website); p53 (mouse, Santa Cruz, sc-126, 1:1,000; Validation: manufacturers website); PARP1 (mouse, Proteintech, 66520-1-Ig, 1:1,000; Validation: manufacturers website); PCNA (mouse, Santa Cruz, sc-56, 1:1,000; Validation: manufacturers website); pH3 (Ser10) (rabbit, Cell Signaling, 53348, 1:1,000; Validation: manufacturers website); RPS3A (rabbit, ABClonal, A5885, 1:1,000; Validation: manufacturers website); RPS6 (mouse, Santa Cruz, sc-74459, 1:1,000; Validation: manufacturers website); SKP1 (rabbit, Cell Signalling, 12248, 1:1,000; Validation: manufacturers website).

Secondary antibody conjugates used for IF were goat anti-rabbit Alexa Fluor 488 (A11034), goat anti-rabbit and goat anti-mouse Alexa Fluor 568 (A11036, A11031), goat anti-rabbit and goat anti-mouse Alexa Fluor 647 (A21245, A21236) (all from Thermo Fischer Scientific, 1:2,000); donkey anti-human Alexa Fluor 647 (Jackson Immuno Research, 709-605-149, 1:2,000); goat anti-mouse (DyLight 488, Thermo Fisher, 35503, 1:1,000); and donkey anti-rabbit IgG (Alexa Fluor® 555, Abcam, ab150070, 1:1,000).

Secondary antibody conjugates used for western blotting were HRP-linked horse anti-mouse IgG (Vector Laboratories, PI-2000, 1:10,000), goat anti-rabbit IgG (Vector Laboratories, PI-1000, 1:10,000), horse anti-mouse IgG (Cell Signaling, 7076, 1:5,000), goat anti-rabbit IgG (Cell Signaling, 7074, 1:5,000) antibodies.

Secondary antibody probes used for proximity ligation assays (PLA) were donkey anti-rabbit PLUS (Sigma-Aldrich, DUO92002) and donkey anti-mouse MINUS (Sigma-Aldrich, DUO92004).

Antibodies used for DNA fibers were:
CldU (anti-BrdU, rat, Abcam, ab6326, 1:200; Validation: previous studies e.g. Sedlackova et al. Nature 2020) and IdU (anti-BrdU, mouse, Becton Dickinson, 347580, 1:200; Validation: previous studies e.g. Sedlackova et al. Nature 2020). Secondary antibodies: goat anti-rat AlexaFluor 594 IgG (Thermo Fisher Scientific, A21209, 1:200), and goat anti-mouse AlexaFluor 488 IgG (Thermo Fisher Scientific, A11029, 1:200).

Validation

Information regarding the validation of antibodies used in this study has been mentioned above (along with the list of antibodies). All the antibodies used in this study were either validated by the manufacturer and/or in previous studies from our or other laboratories.

Eukaryotic cell lines

Policy information about cell lines and Sex and Gender in Research

Cell line source(s)	Parental U2OS cell (ATCC HTB-96); its derivatives used in this study:<br>CDC45-mEGFP, MCM4-HALO;<br>MCMBP-KO, MCM4-HALO (used as parental cell line to generate doxycycline-inducible wt/C-terminal deleted (ΔC) MCMBP variants);<br>DCAF12-KO#1 (used as parental cell line to generate doxycycline-inducible Strep-DCAF12);<br>DCAF12-KO#2 (used as parental cell line to generate doxycycline-inducible Strep-DCAF12).<br><br>RPE-1: hTERT-RPE1 (ATCC, CRL-4000).<br><br>Parental HEK293T (ATCC, CRL-1573) was used to generate cell lines transiently expressing Strep II-FLAG-tagged CCNF/FBXL18/FBXO28/FBXO38/DCAF8/DCAF12 or HA-tagged MOV10, MCMBP, or their C-terminal deletion mutants.<br><br>The origin and generation of derivatives of parental HCT116 (ATTC, CCL-247) ectopically expressing Strep-DCAF12/Strep-DCAF12(Δ1-11)/Strep-DCAF12(Δ1-38) was described in Lidak et al. Int J Mol Sci 2021.
Authentication	The parental U2OS, hTERT-RPE1, HEK293T and HCT116 cell lines were authenticated by STR profiling. No further authentication of cell lines was performed.
Mycoplasma contamination	All the cell lines were routinely tested (monthly basis) for mycoplasma (Mycoplasma Detection Kit; InvivoGen, rep-mys-50) and always found negative.
Commonly misidentified lines<br>(See ICLAC register)	No commonly misidentified cell lines were used in this study.

Plants

Seed stocks	Not applicable, because this study does not involve plants.
Novel plant genotypes	Not applicable, because this study does not involve plants.
Authentication	Not applicable, because this study does not involve plants.
