# Supplementary material for: CRL4DCAF12 regulation of MCMBP ensures optimal licensing of DNA replication
Source: Nat Commun. 2025 Oct 27;16:9391. doi: 10.1038/s41467-025-64258-5 (PMC12559739; doi:10.1038/s41467-025-64258-5)
Supplement: Supplementary file 4 — Source Data [file 41467_2025_64258_MOESM4_ESM.zip › Yadav_Abdirov_et_al_Source data_02092025/Uncropped scans of all bots and gels.pdf]

Figure 1j

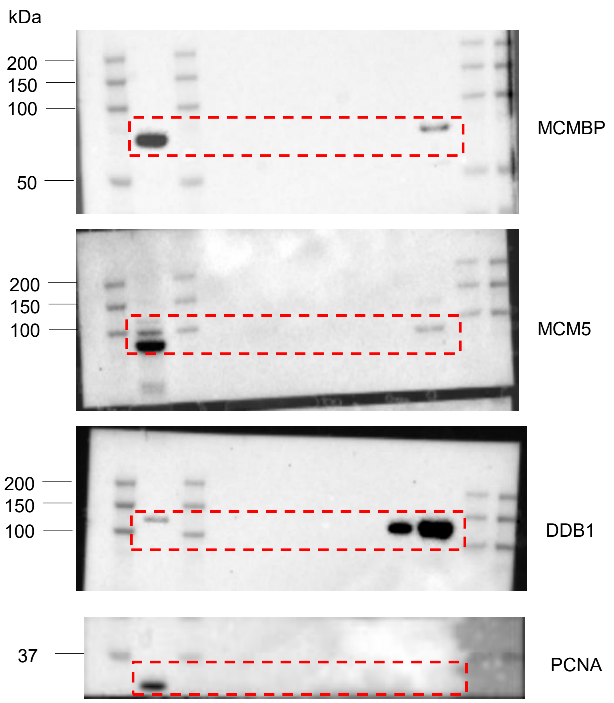

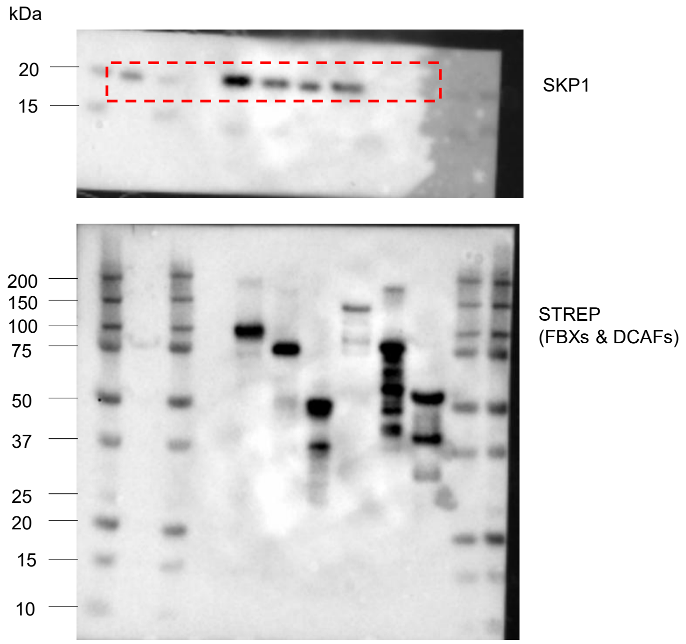

Presented membranes represent merge images of chemiluminescence and digital channels.

Figure 1k

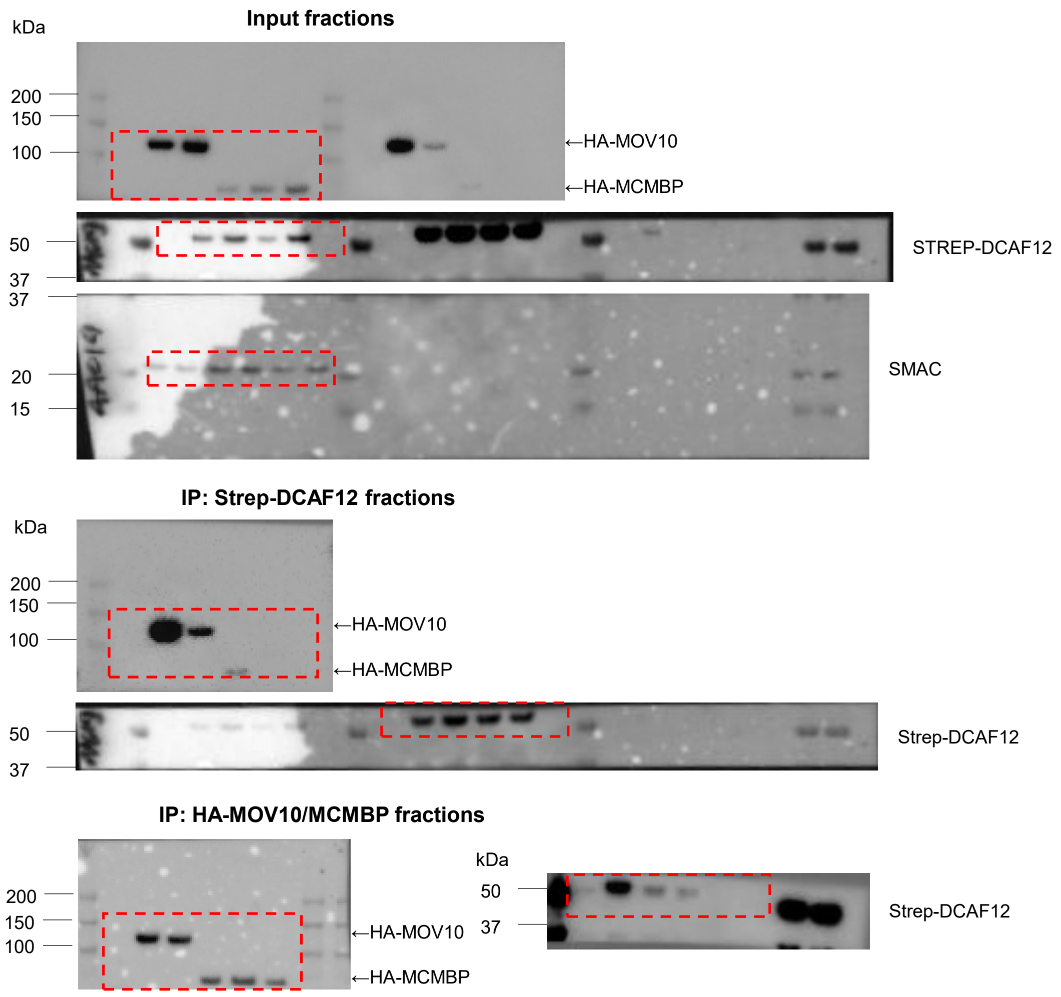

Presented membranes represent merge images of chemiluminescence and digital channels.

Figure 2e

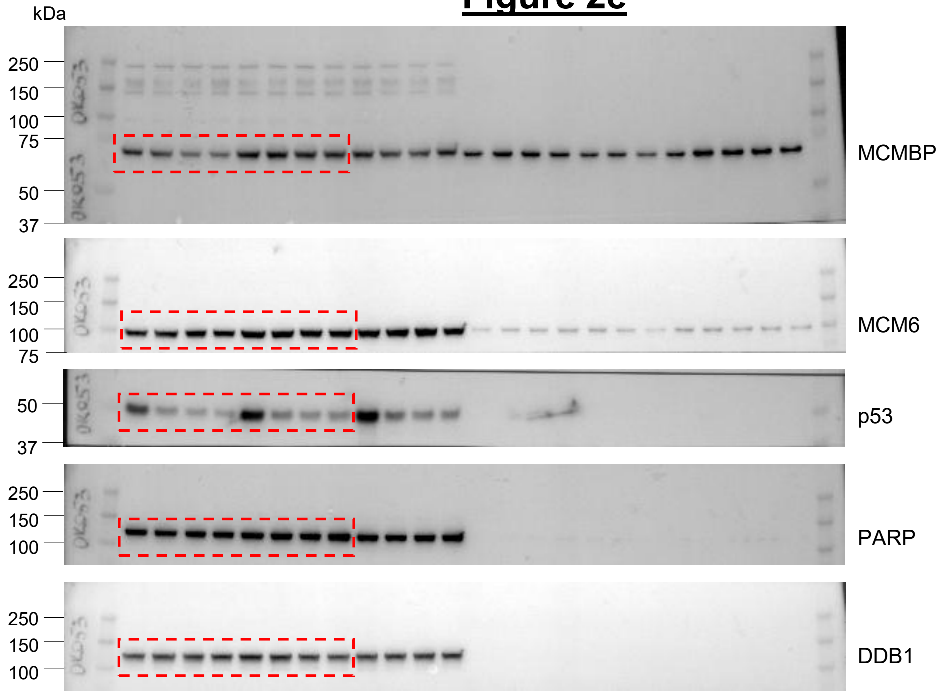

Presented membranes represent merge images of chemiluminescence and digital channels.

Figure 2e – Biological replicate

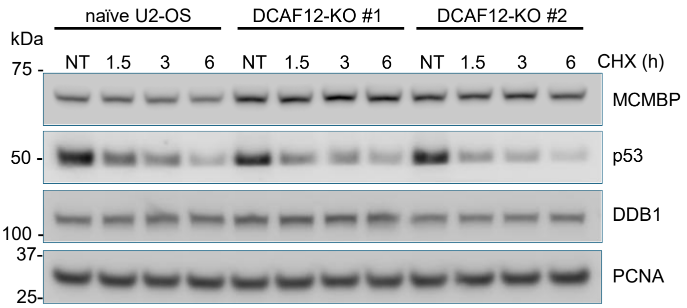

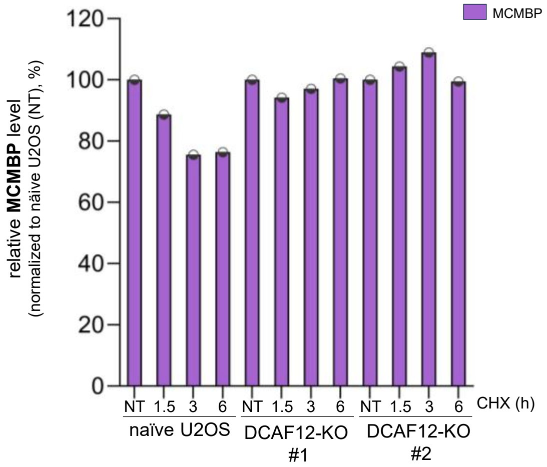

Densitometry of MCMBP based on western blots in left

Figure 2e – Biological replicate (uncropped blots)

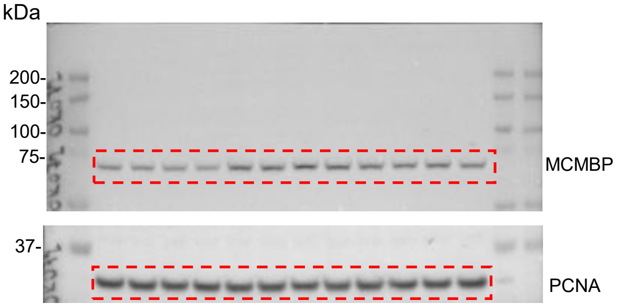

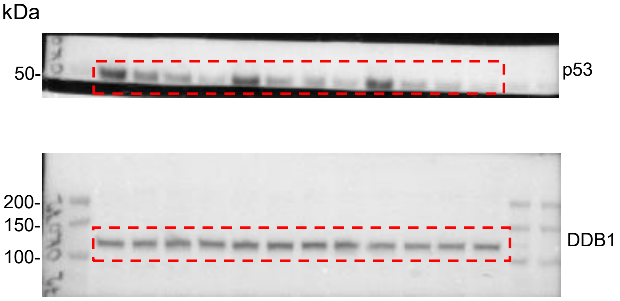

Presented membranes represent merge images of chemiluminescence and digital channels.

Figure 2h

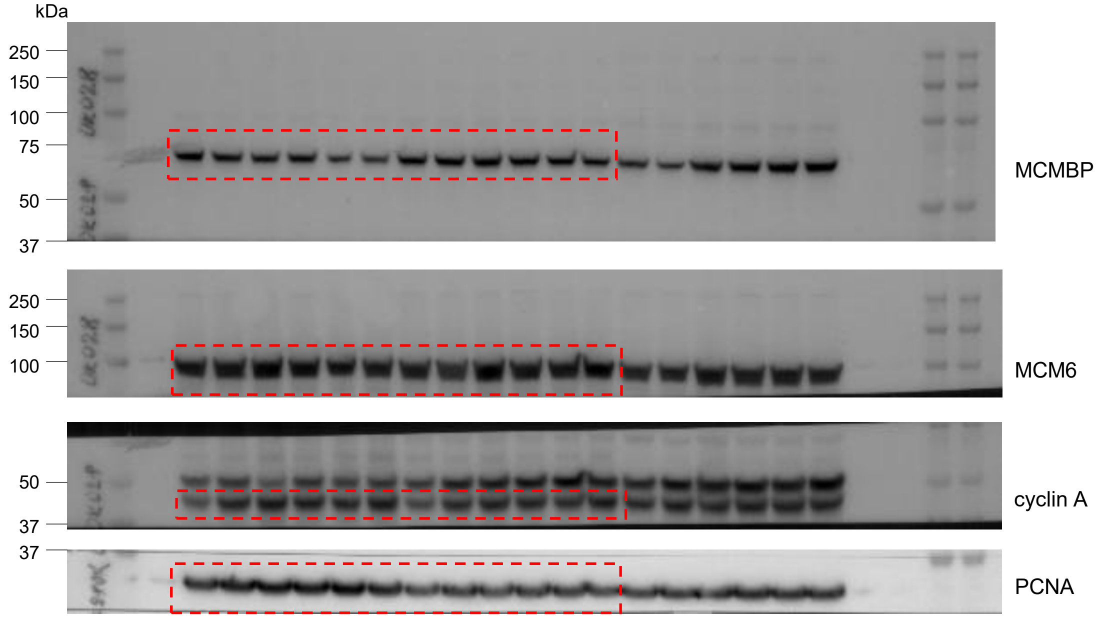

Presented membranes represent merge images of chemiluminescence and digital channels.

Figure 2h – Biological replicate

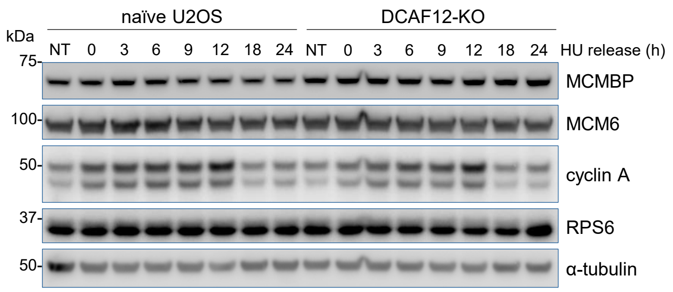

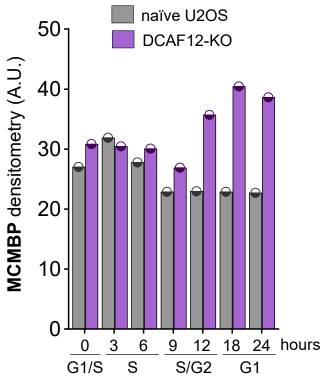

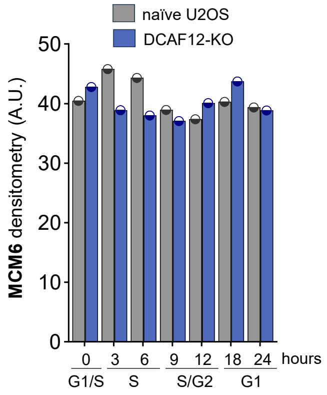

Densitometry of selected proteins based on western blots in left

Figure 2h – Biological replicate (uncropped blots)

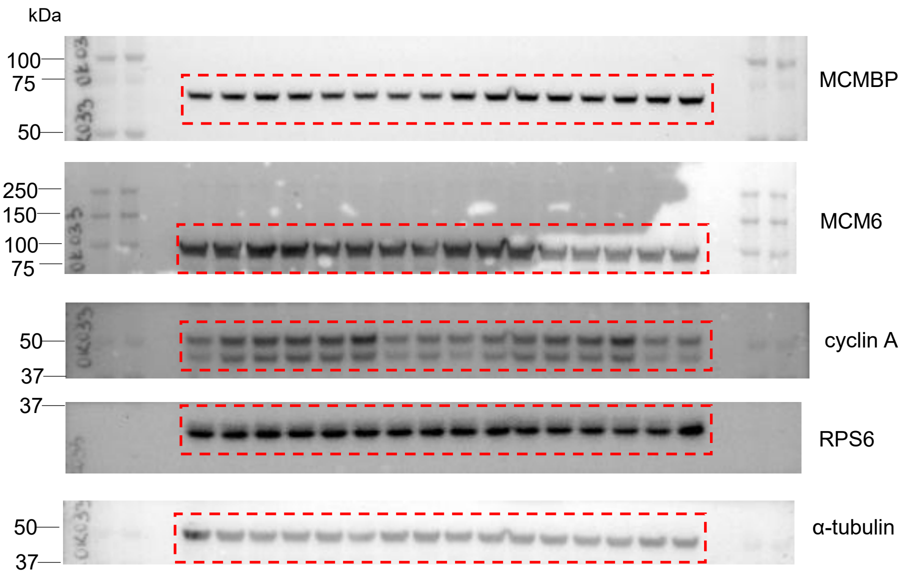

Presented membranes represent merge images of chemiluminescence and digital channels.

Figure 2j

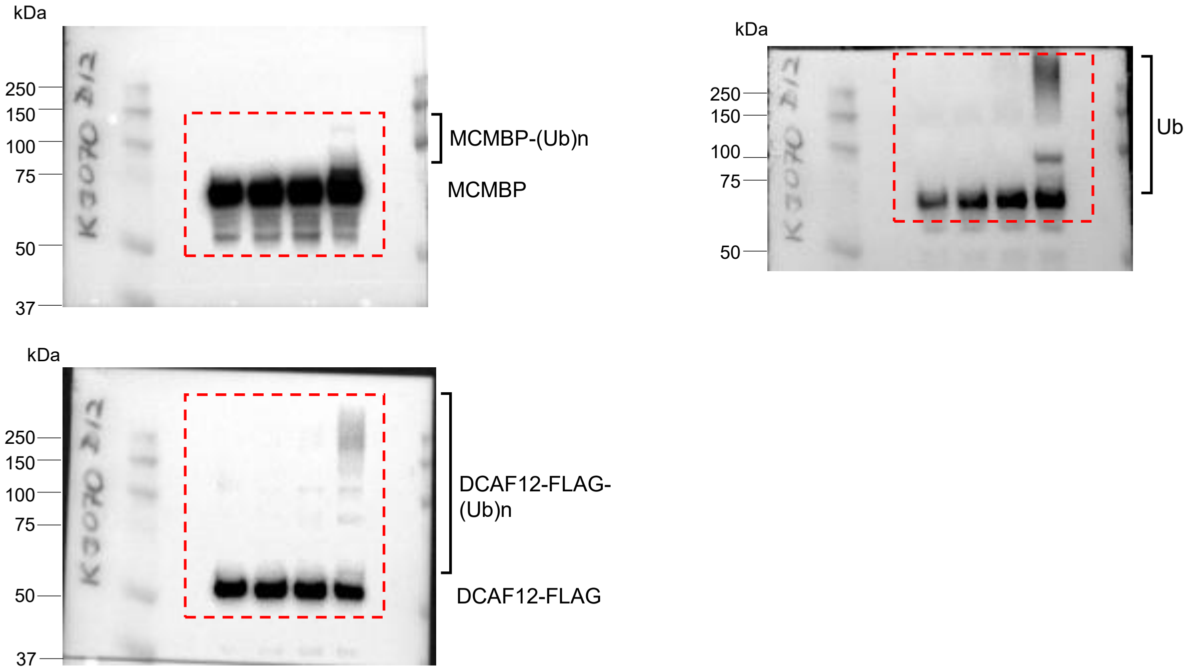

Presented membranes represent merge images of chemiluminescence and digital channels.

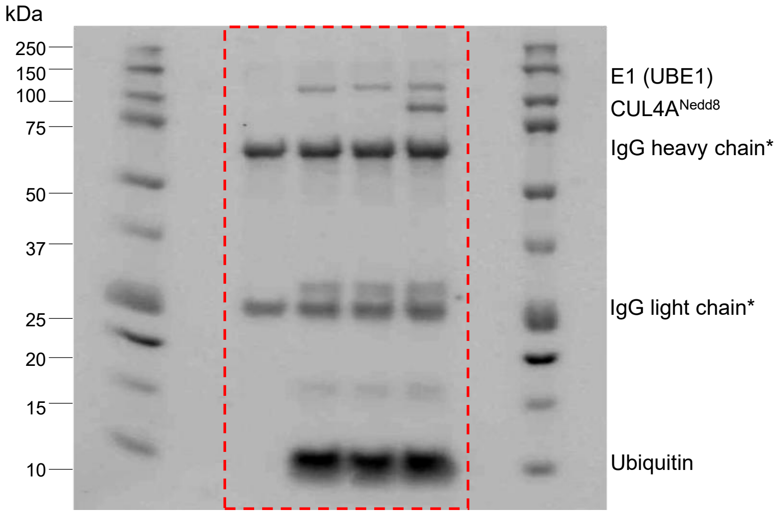

PonceauS

Figure 5b

MCM4-Halo IP (*150 mM NaCl*) – Biological Replicate #1 (uncropped blots)

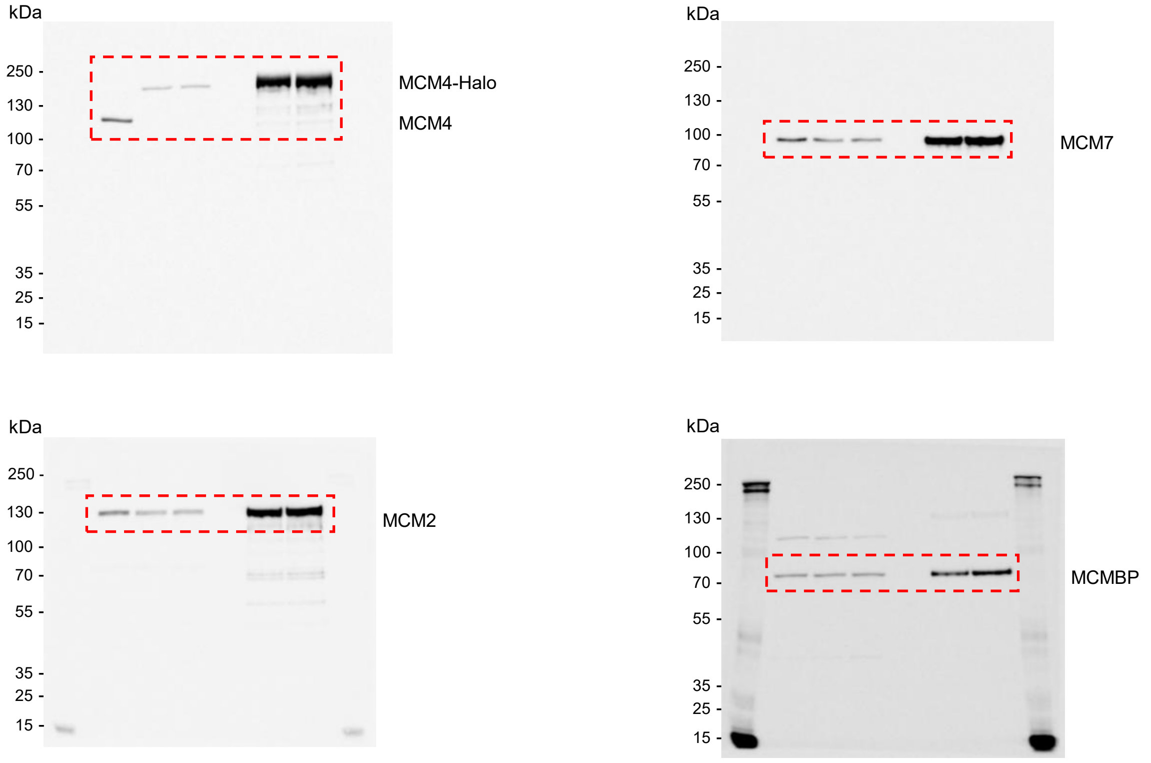

Figure 5b

MCM4-Halo IP (*150 mM NaCl*) – Biological Replicate #2

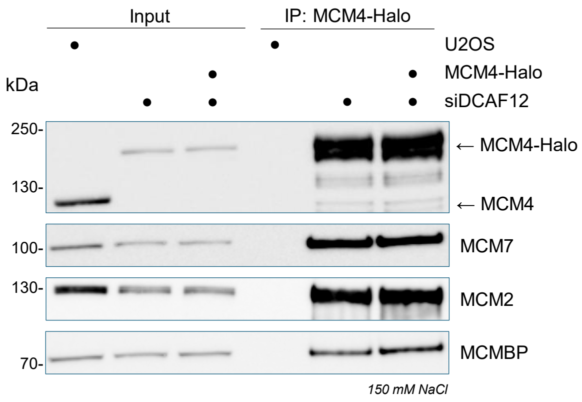

MCM4-Halo IP (*150 mM NaCl*) – Biological Replicate #2 (uncropped blots)

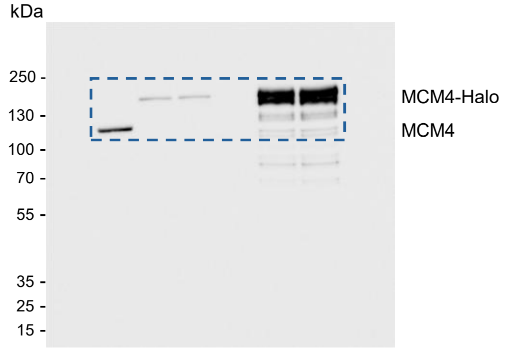

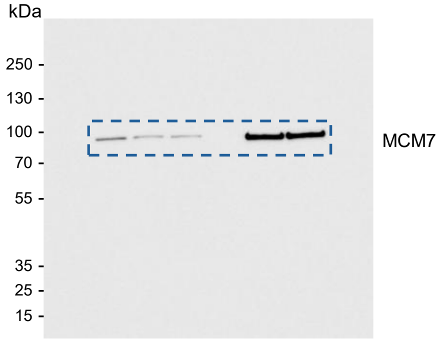

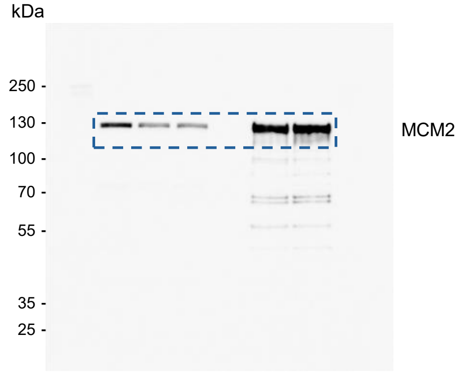

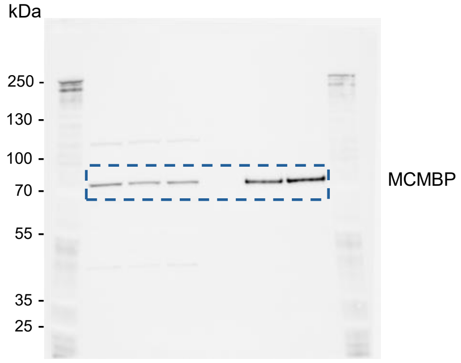

Figure 5d

MCM4-Halo IP (nuclear fraction only, 150 mM NaCl) – Biological Replicate #1 (uncropped blots)

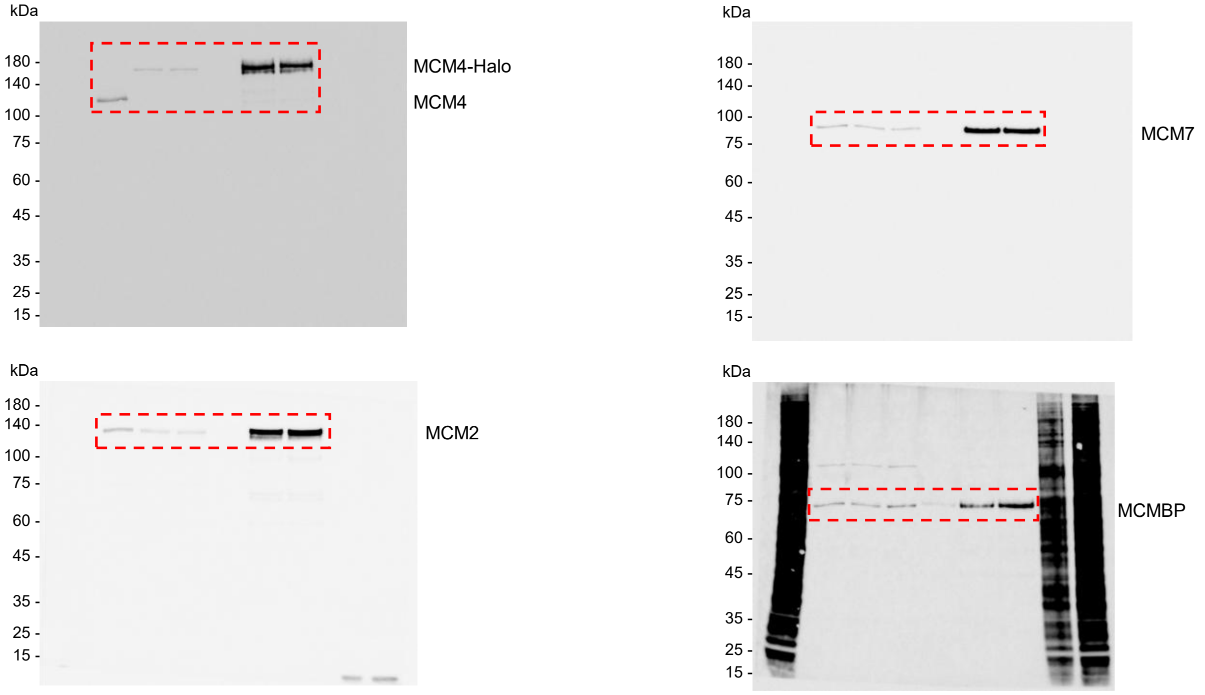

Figure 5d

MCM4-Halo IP (nuclear fraction only, 150 mM NaCl) – Biological Replicate #2

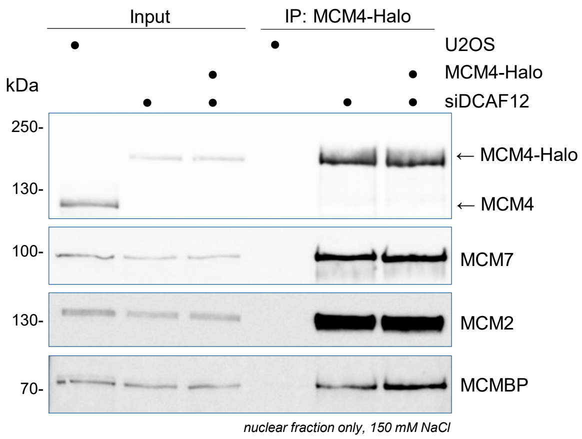

MCM4-Halo IP (nuclear fraction only, 150 mM NaCl) – Biological Replicate #2 (uncropped blots)

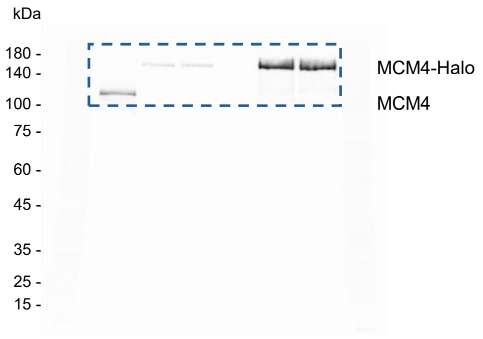

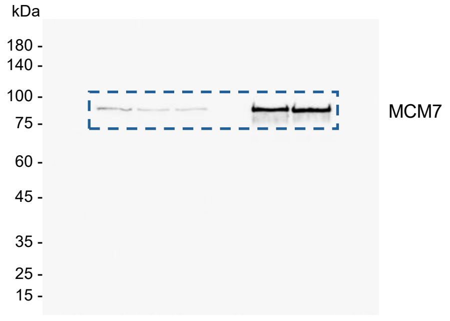

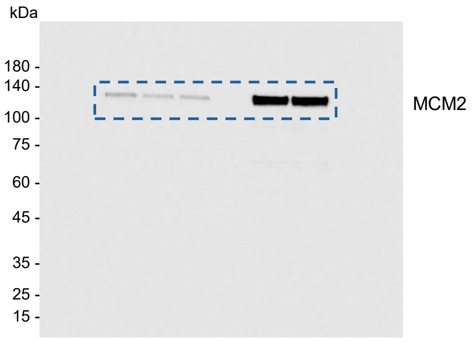

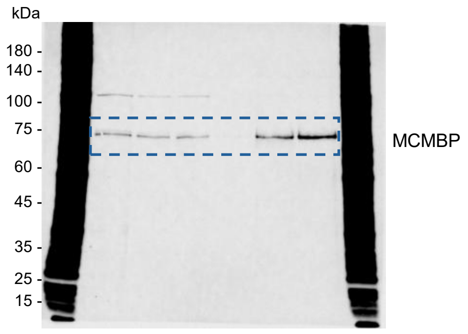

Supplementary Figure 2i

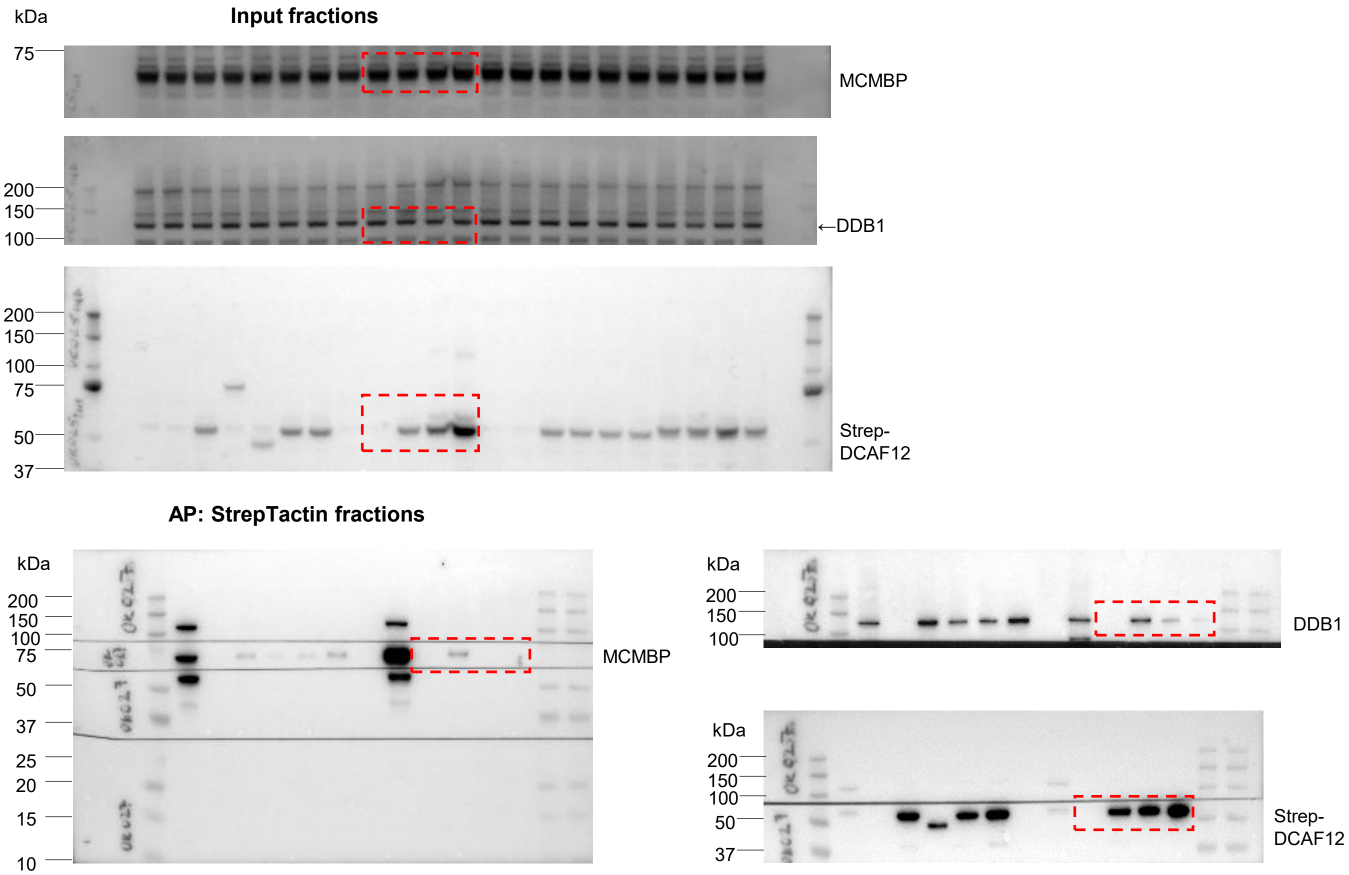

Presented membranes represent merge images of chemiluminescence and digital channels.

Supplementary Figure 3k

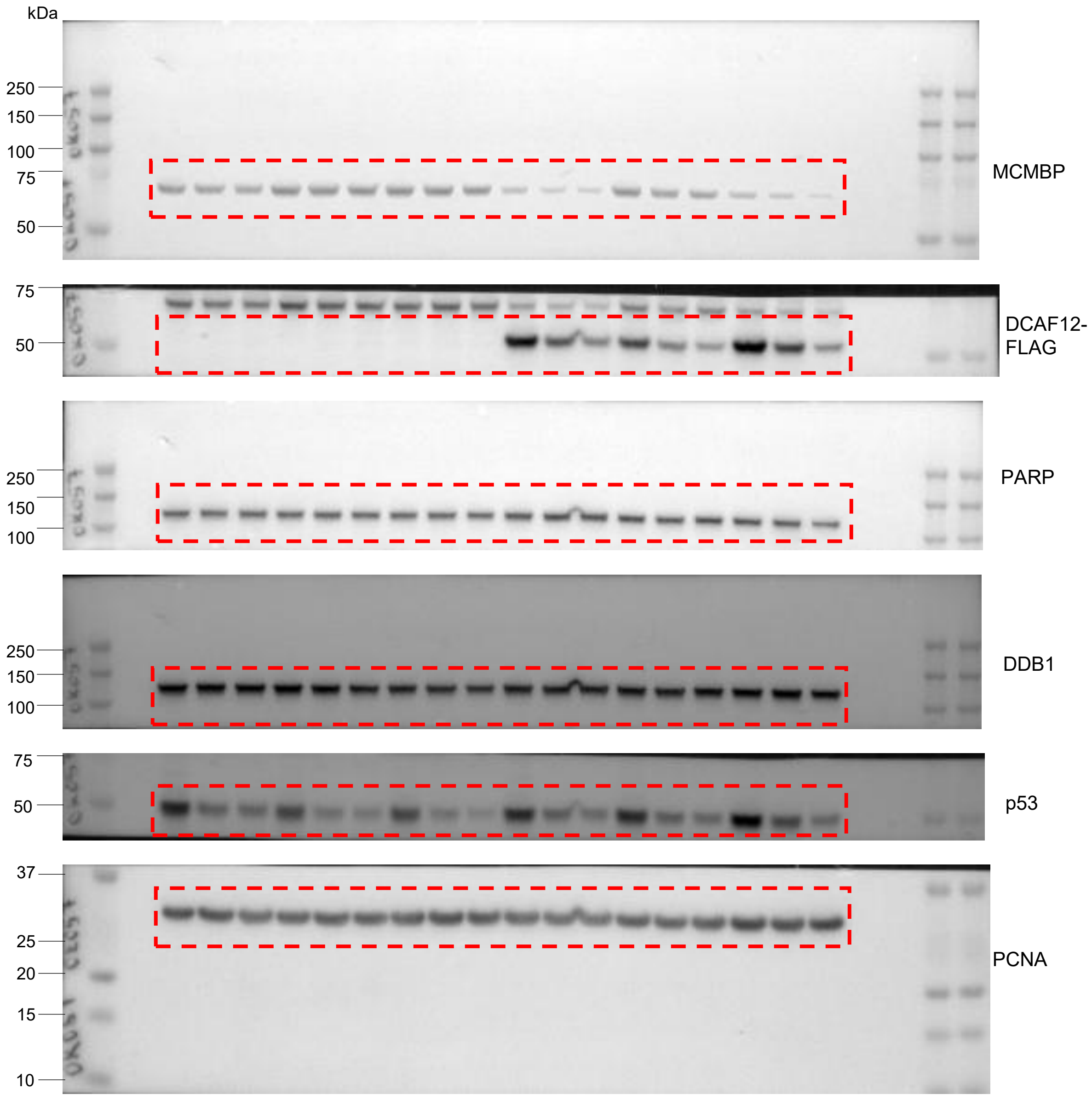

Presented membranes represent merge images of chemiluminescence and digital channels.

Supplementary Figure 6a

MCM4-Halo IP (*500 mM NaCl*) – Biological Replicate #1 (uncropped blots)

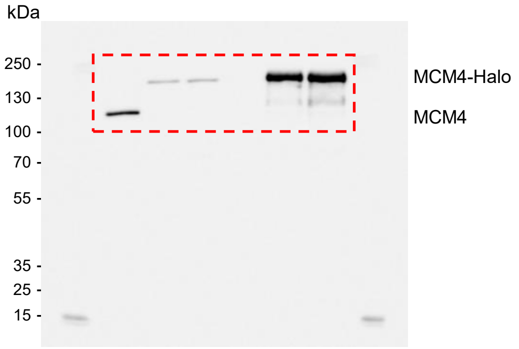

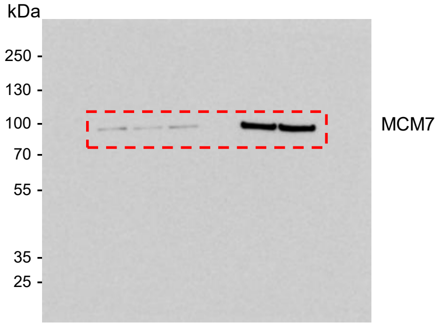

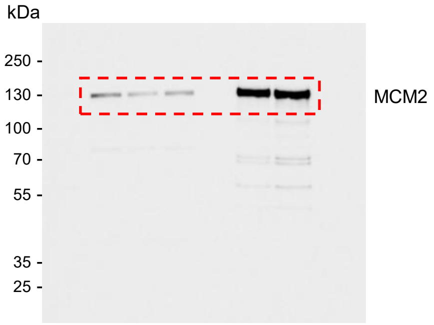

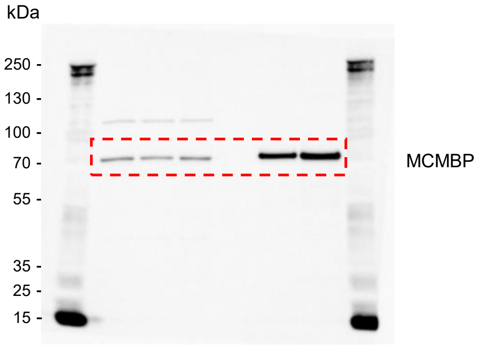

Supplementary Figure 6a

MCM4-Halo IP (*500 mM NaCl*) – Biological Replicate #2

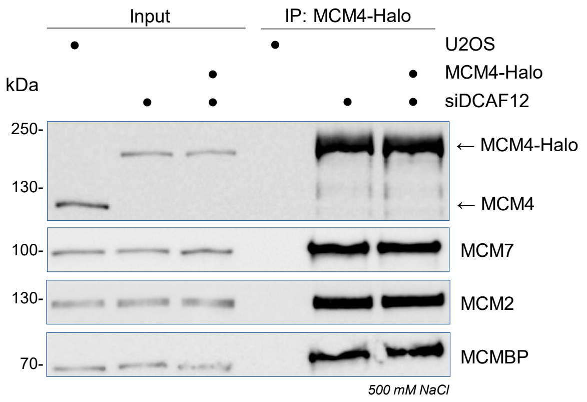

MCM4-Halo IP (*500 mM NaCl*) – Biological Replicate #2 (uncropped blots)

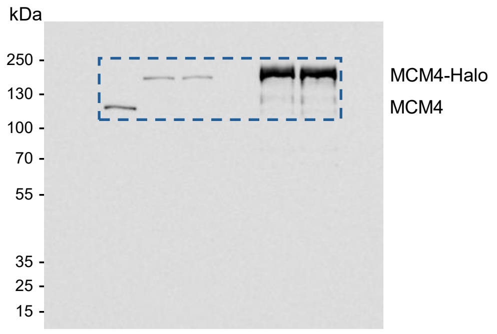

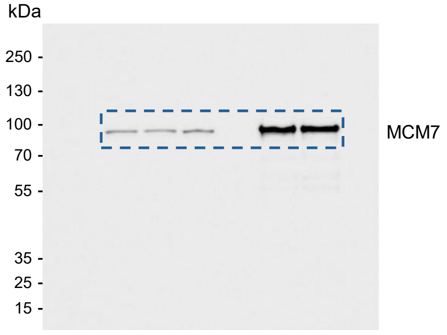

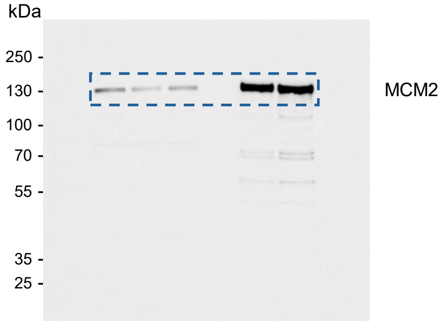

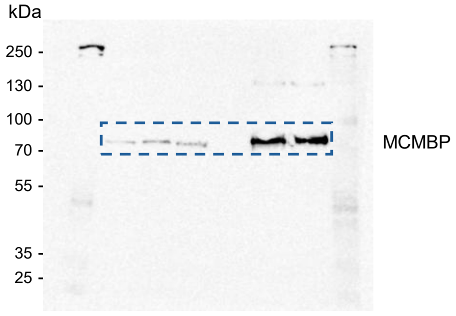

Supplementary Figure 6d

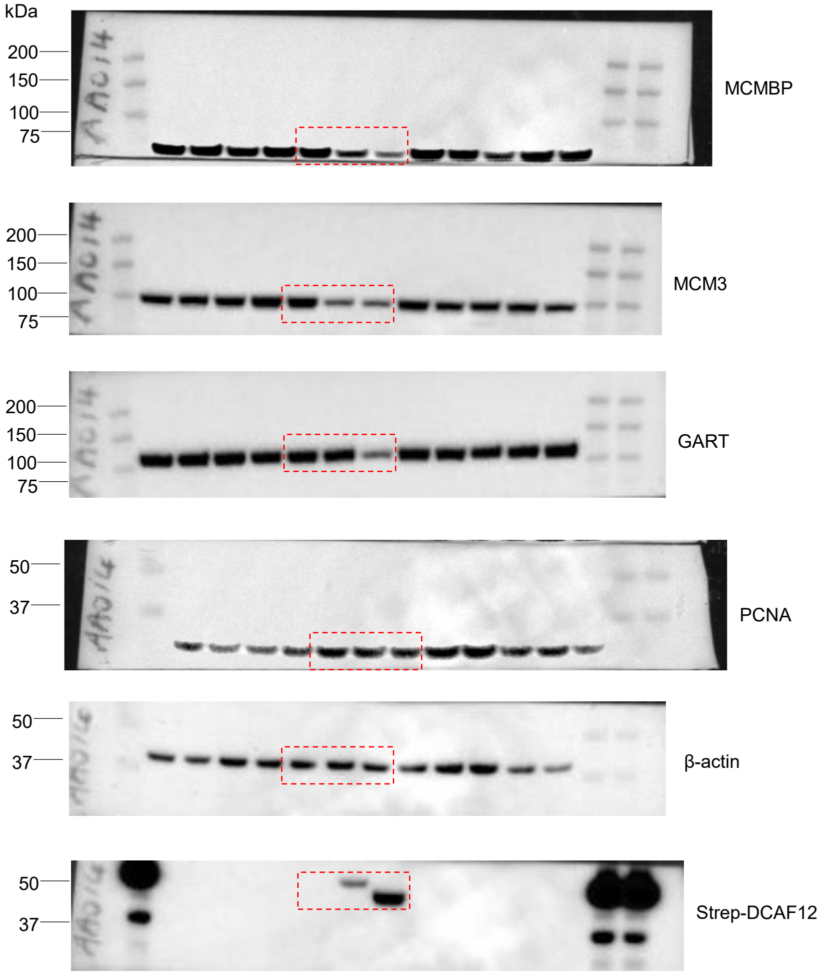

Supplementary Figure 6g

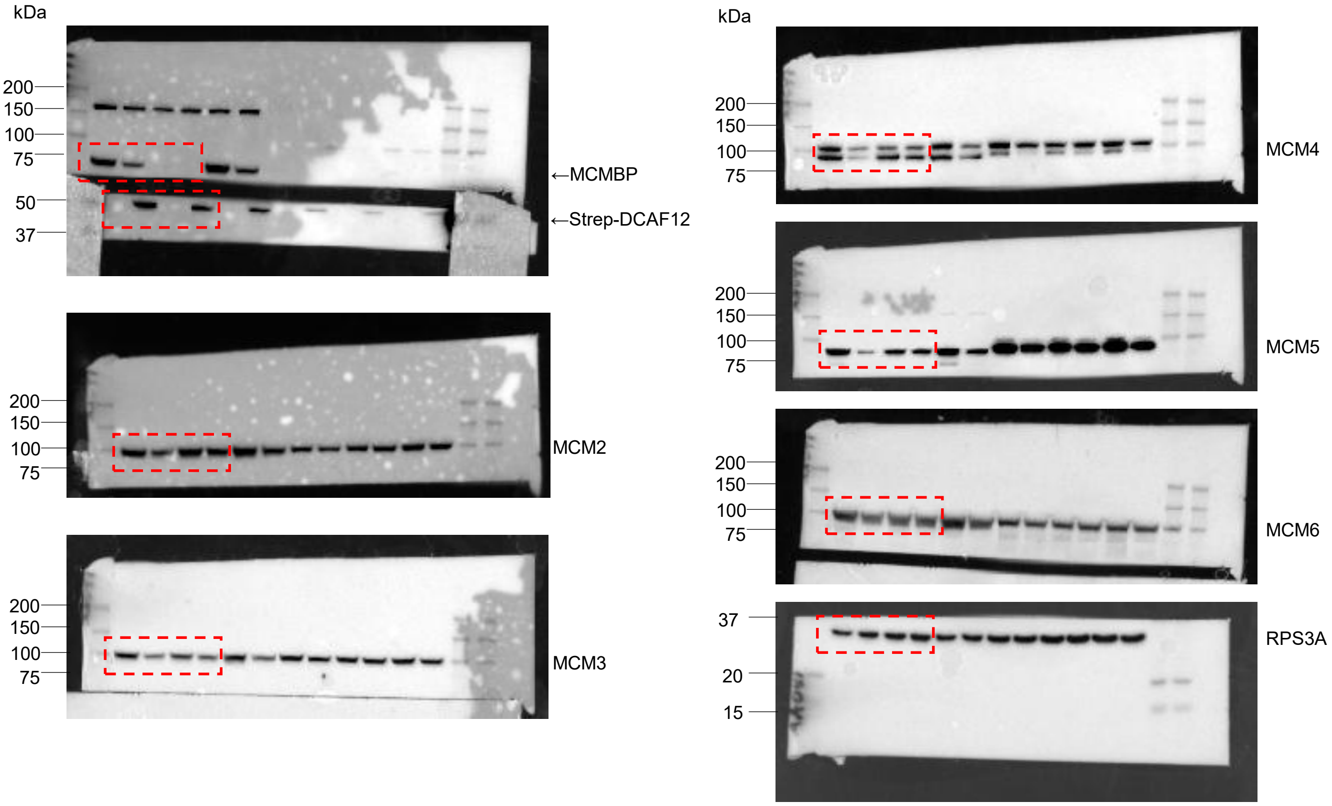

Presented membranes represent merge images of chemiluminescence and digital channels.

Validation of antibodies used in Supplementary Fig. 4

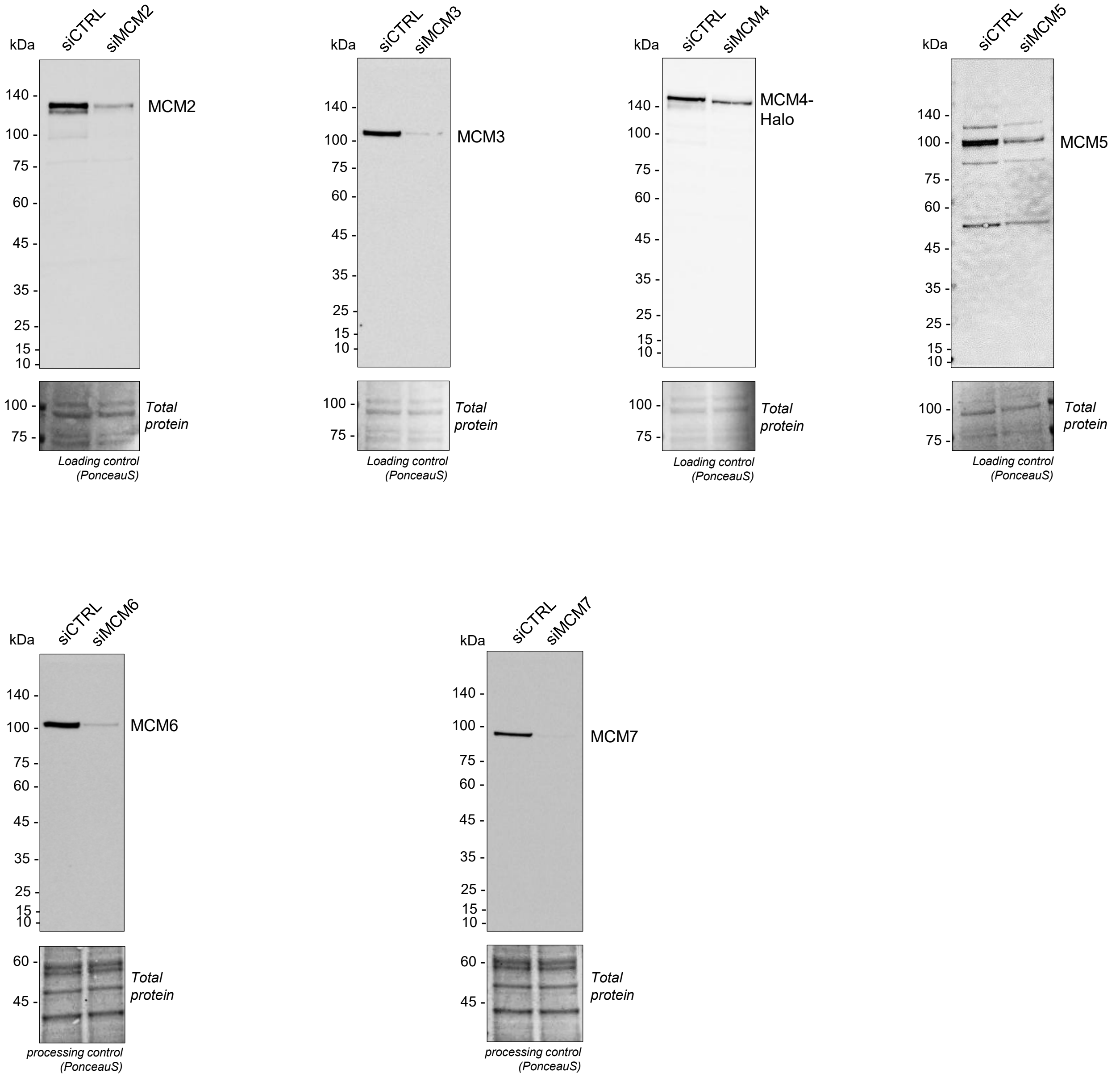

siRNAs used for validation of antibodies in **Supplementary Fig. 4** were as follows: MCM2 (Ambion, s8587); MCM3 (Ambion, s8589); MCM4 (Ambion, s8592); MCM5 (Thermo Fisher Scientific, 4390827); MCM6 (Ambion Silencer Select: s8598); MCM7 (Ambion Silencer Select: s224035)

Validation of antibodies used in Supplementary Fig. 4 (uncropped blots)

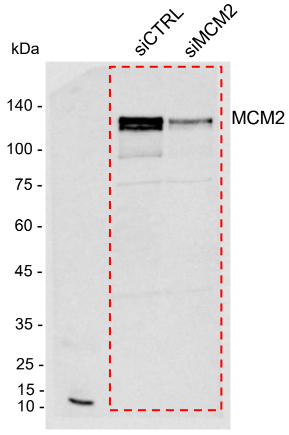

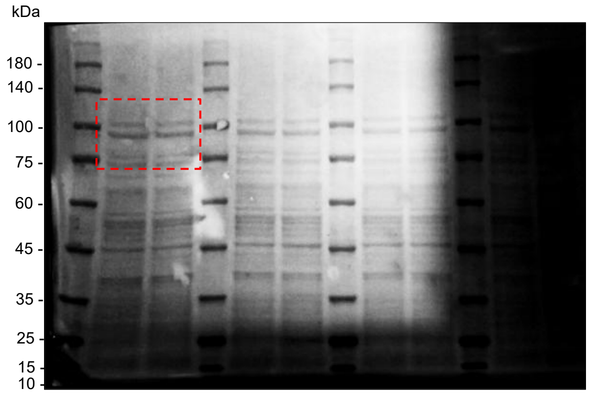

Loading control (PonceauS)

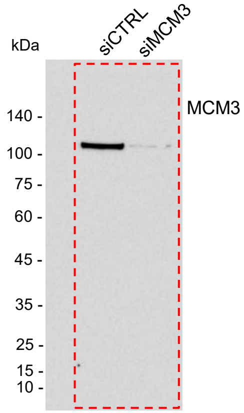

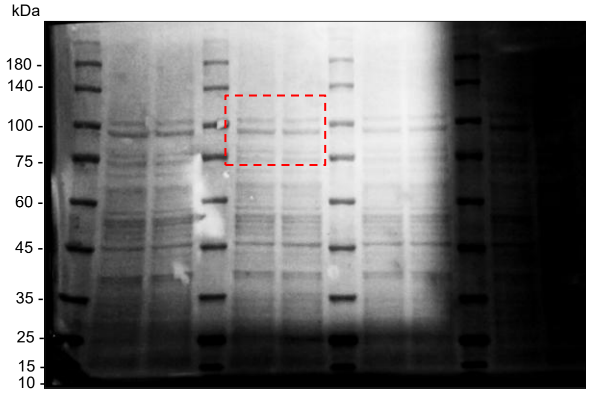

Loading control (PonceauS)

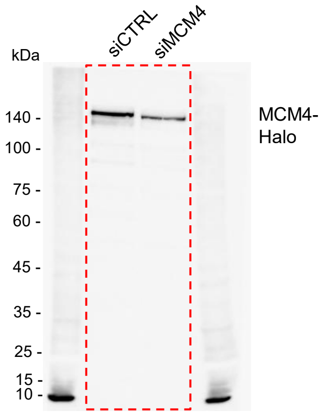

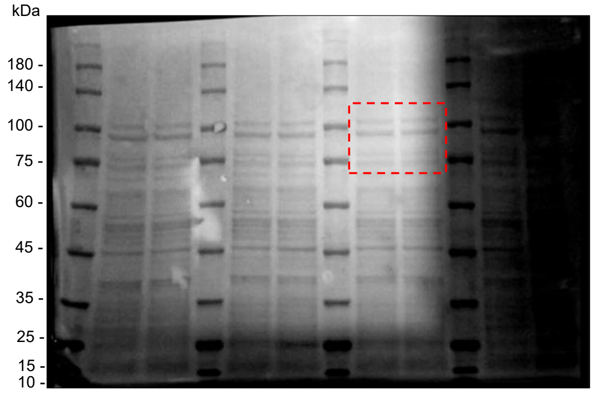

Loading control (PonceauS)

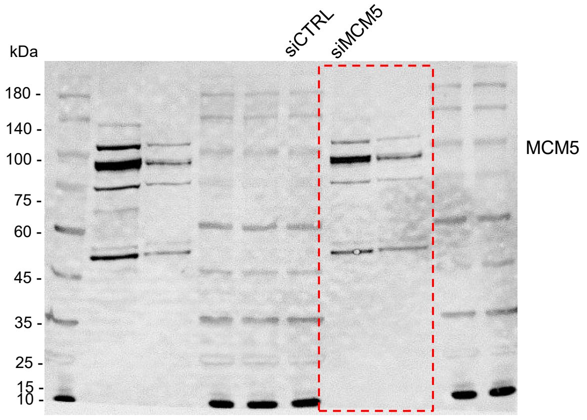

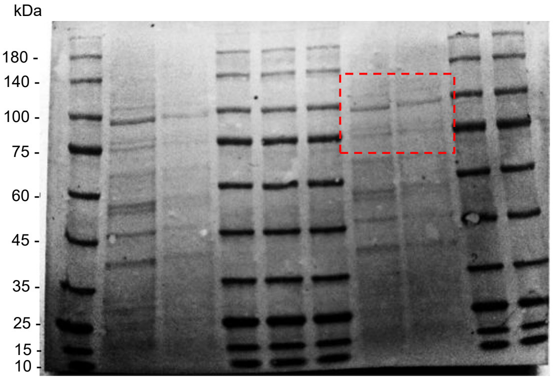

Loading control (PonceauS)

Validation of antibodies used in Supplementary Fig. 4 (uncropped blots)

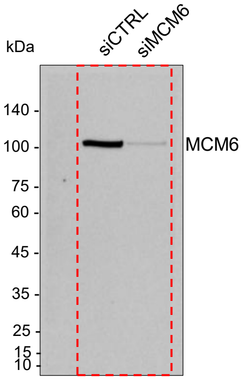

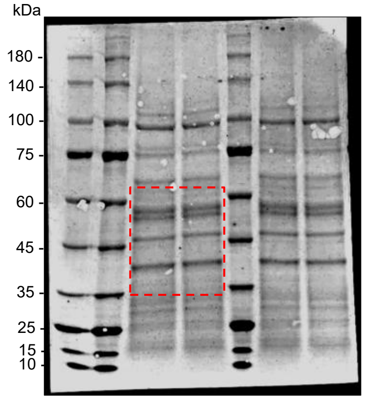

processing control (PonceauS)

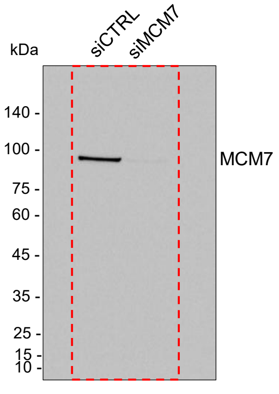

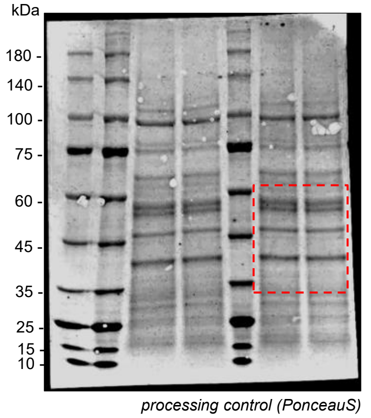

processing control (PonceauS)
